# Bacteriophage therapy against pathological *Klebsiella pneumoniae* ameliorates the course of primary sclerosing cholangitis

**DOI:** 10.1038/s41467-023-39029-9

**Published:** 2023-06-05

**Authors:** Masataka Ichikawa, Nobuhiro Nakamoto, Sharon Kredo-Russo, Eyal Weinstock, Iddo Nadav Weiner, Efrat Khabra, Noa Ben-Ishai, Dana Inbar, Noga Kowalsman, Ron Mordoch, Julian Nicenboim, Myriam Golembo, Naomi Zak, Jagoda Jablonska, Hila Sberro-Livnat, Sharon Navok, Nufar Buchshtab, Takahiro Suzuki, Kentaro Miyamoto, Toshiaki Teratani, Sota Fujimori, Yoshimasa Aoto, Mikiko Konda, Naoki Hayashi, Po-Sung Chu, Nobuhito Taniki, Rei Morikawa, Ryosuke Kasuga, Takaya Tabuchi, Shinya Sugimoto, Yohei Mikami, Atsushi Shiota, Merav Bassan, Takanori Kanai

**Affiliations:** 1 Division of Gastroenterology and Hepatology, Department of Internal Medicine, Keio University, Tokyo, Japan; 2 BiomX Ltd., Ness Ziona, Israel; 3 JSR-Keio University Medical and Chemical Innovation Center (JKiC), JSR Corp., Tokyo, Japan; 4 Department of Microbiology and Immunology, Keio University, Tokyo, Japan; 5 Microbiopharm Japan, Co. Ltd., Tokyo, Japan; 6 Japan Agency for Medical Research and Development, AMED, Tokyo, Japan

**Keywords:** Primary sclerosing cholangitis, Dysbiosis

## Abstract

Primary sclerosing cholangitis (PSC) is characterized by progressive biliary inflammation and fibrosis. Although gut commensals are associated with PSC, their causative roles and therapeutic strategies remain elusive. Here we detect abundant *Klebsiella pneumoniae* (Kp) and *Enterococcus gallinarum* in fecal samples from 45 PSC patients, regardless of intestinal complications. Carriers of both pathogens exhibit high disease activity and poor clinical outcomes. Colonization of PSC-derived Kp in specific pathogen-free (SPF) hepatobiliary injury-prone mice enhances hepatic Th17 cell responses and exacerbates liver injury through bacterial translocation to mesenteric lymph nodes. We developed a lytic phage cocktail that targets PSC-derived Kp with a sustained suppressive effect in vitro. Oral administration of the phage cocktail lowers Kp levels in Kp-colonized germ-free mice and SPF mice, without off-target dysbiosis. Furthermore, we demonstrate that oral and intravenous phage administration successfully suppresses Kp levels and attenuates liver inflammation and disease severity in hepatobiliary injury-prone SPF mice. These results collectively suggest that using a lytic phage cocktail shows promise for targeting Kp in PSC.

## Abstract (web summary)

Patients with primary sclerosing cholangitis (PSC) harboring *Klebsiella pneumoniae* (Kp) exhibit poor clinical outcomes. Here, the authors show that administration of a phage cocktail targeting PSC-derived Kp reduces bacterial burden in Kp-colonized mice and alleviates liver injury.

## introduction

Primary sclerosing cholangitis (PSC) is an idiopathic, heterogeneous, cholestatic liver disease characterized by persistent progressive biliary inflammation and fibrosis that leads to liver cirrhosis and end-stage liver disease^1,2^. No effective medical therapy has been developed for PSC other than liver transplantation. In approximately 60–80% of patients in western countries, a key clinical characteristic of PSC is comorbidity with inflammatory bowel disease (IBD), mostly ulcerative colitis (UC) and more rarely, Crohn’s disease or IBD unclassified^1,3^. In addition, mounting evidence has indicated that the intestinal microbiota plays a role in PSC pathogenesis^4,5^. However, it remains uncertain whether comorbidity with colitis or disease-associated bacteria in the microbiome affects the clinical course of PSC.

In our recent study, we reported the frequent presence of specific gut microbes, namely, *Klebsiella pneumoniae* (Kp), *Proteus mirabilis* (Pm), and *Enterococcus gallinarum* (Eg), in fecal samples obtained from patients with PSC + IBD in our Japanese cohort^6^. These results are consistent with those of similar reports from a European cohort^7^. Notably, the combination of these three gut bacteria derived from patients with PSC + IBD directly contributed to the pathogenesis of the hepatobiliary phenotype^6^. Mechanistically, using gnotobiotic mice, we found that these bacteria translocated to mesenteric lymph nodes and contributed to subsequent T helper 17 (Th17) cell induction in the liver. However, it remains unclear whether these findings apply to PSC patients without IBD, whether the abundance of these microbiota is associated with the clinical outcome of PSC, and whether specific bacteria could serve as effective therapeutic targets in a clinical setting.

In an attempt to examine the possibility that manipulation of the gut microbiota may prevent or treat PSC, researchers have previously demonstrated partial improvement in the levels of serum hepatobiliary enzymes following short-term administration of antibiotics^8^ or fecal microbiota transplantation from healthy individuals^9^. However, the long-term effects of these therapies on the suppression of liver inflammation and fibrosis have not been clarified. As an alternative therapeutic tool for targeting specific gut microbiota, increasing attention has been paid to the use of bacteriophages. Bacteriophages, also known as phages, are self-replicating viruses that infect bacteria with high host specificity^10^. Lytic phages attach to bacterial cells via cognate bacterial receptors and inject their viral genome into the host cell. The phage genes exploit the host gene expression machinery, leading to the replication, synthesis, and assembly of phage progenies. Finally, the bacterial cell is lysed by phage products, thus releasing the phage progenies into the environment^11–14^. Recent interest in the systemic use of phage therapy has shown promising results against various pathobionts^15–17^. Furthermore, the safety and potential for oral delivery of phage targeting Kp have been recently shown in humans^18^. However, to date, limited data are available, which would support the sustained efficacy of phage for treating hepatobiliary diseases and the feasibility of this approach in humans. Here, we show that a phage cocktail specifically targeting a PSC-derived Kp strain exhibits sustained suppressive effects in vitro and reduces Kp levels in vivo.

## Results

### Colonization by specific gut pathobionts is associated with the clinical course of PSC

**Fig. 1:**
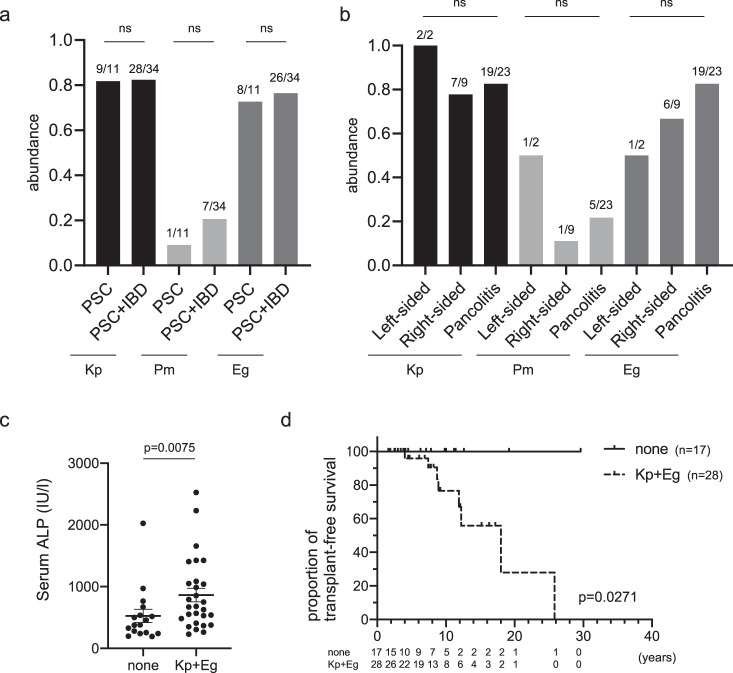
The gut microbiota is associated with the clinical course of PSC. **a**, **b** The prevalence of Kp, Pm, and Eg in fecal samples of patients with PSC was classified according to the presence of IBD complications (**a**) and the locations affected by colitis (**b**). Fisher’s exact test (**a**) or Chi-square test (**b**) were applied to compare the differences; ns not significant. **c**, **d** Characteristics of patients with PSC carrying both Kp and Eg compared with those of non-carriers. **c** Serum ALP levels. The data shown represent the mean ± standard error of the mean (SEM) (non-carriers: *n* = 17 patients, Kp+Eg carriers: *n* = 28 patients). The two-sided Mann–Whitney test was applied. **d** Transplant-free survival (non-carriers: *n* = 17 patients, Kp+Eg carriers: n = 28 patients). Kaplan–Meier analysis was applied to determine the cumulative survival percentages, and differences between groups were compared using the two-sided log-rank test.

We collected fecal samples from a cohort of 45 patients with PSC, either complicated with IBD (*n* = 34) or without IBD (*n* = 11) in a cohort following obtaining informed consent and examined associations between clinical hepatobiliary parameters and the clinical course. The clinical characteristics of the patients are summarized in Supplementary Table 1. We first confirmed that the abundance of the three PSC-associated gut microbiota did not differ between patients with PSC and patients with PSC + IBD (Kp: PSC, 82% vs. PSC + IBD, 82%; Pm: PSC, 9% vs. PSC + IBD, 21%; and Eg: PSC, 73% vs. PSC + IBD, 76%; Fig. 1a), which was consistent with the results of our previous report^6^. Interestingly, the distribution of colitis (right-sided, left-sided, or pancolitis) did not affect the abundance of the microbiota (Fig. 1b). We initially hypothesized that the levels of these bacteria affect the levels of serum hepatobiliary enzymes; however, no significant correlation was found between the abundance of each bacterium separately and serum alkaline phosphatase (ALP) levels (Supplementary Fig. 1a). Subsequently, we investigated whether carriage of each bacterium or its combination could affect the clinical course. Patients carrying both Kp and Eg showed higher serum ALP levels at the time of fecal collection than did the non-carrier group (Fig. 1c, Supplementary Fig. 1b, and Supplementary Table 2), which aligns with previous findings from gnotobiotic mice, indicating that the combination of Kp and Eg triggered inflammatory responses in the liver^6^. Moreover, we discovered that carriers of both Kp and Eg had poorer transplant-free survival than did non-carriers (Fig. 1d and Supplementary Table 2).

### Phage combinations against Kp derived from a patient with PSC efficiently suppressed bacterial growth in vitro

**Fig. 2:**
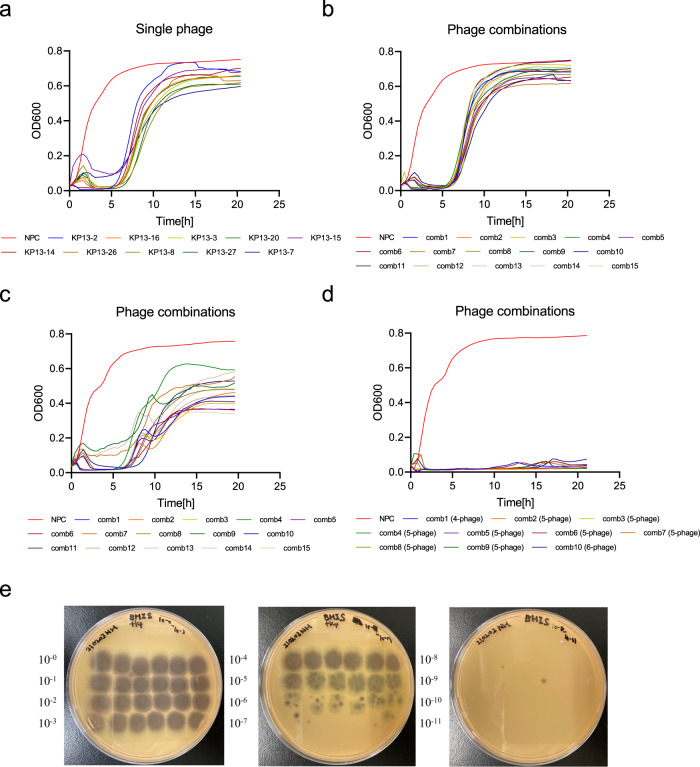
Phage combinations against Kp derived from patients with PSC efficiently suppressed bacterial growth in vitro. **a**–**d** Testing the in vitro-growth suppression by single phages (**a**) and phage combinations (**b**–**d**) against Kp-P1. Bacterial growth was assessed by determining the OD_600_ of 0.2 over a 20 h period. The results obtained with the final optimized phage cocktail are shown in **d**. Each plot shows the median of three biologically independent samples. **e** Representative photo of the plaque assay demonstrating bacteriolysis observed with serially diluted concentrations of the selected phage cocktail.

To directly target specific members of the gut microbiota, we investigated the potential clinical application of bacteriophages against a Kp strain (Kp-P1) isolated from a patient with PSC. Using gnotobiotic mice, we have previously shown that this Kp strain damages the colonic epithelium, promotes bacterial translocation, and increases susceptibility to Th17-mediated hepatobiliary injury^6^. Phages targeting Kp-P1 were isolated and tested iteratively until the development of a phage combination (phage cocktail) that could suppress the regrowth of phage-resistant mutants in vitro for at least 20 h. Initially, we recovered 25 phages against Kp-P1 from processed sewage samples using direct isolation or enrichment, followed by isolation using double-layer spot assays. The phages were sequenced to determine their purity and were assigned to a genomic cluster, based on their similarity to each other. Ten representative phages were selected based on the clustering analysis (KP13-2, KP13-3, KP13-7, KP13-8, KP13-14, KP13-15, KP13-16, KP13-20, KP13-26, and KP13-27). Each of the single phages demonstrated transient growth inhibition of Kp-P1 in liquid culture with the emergence of resistant bacteria (Fig. 2a). Combinations of two to five phages from different clusters were generated and similarly tested to examine their infection dynamics (Methods) with resistant bacteria emerging after ~6 h following administration of all cocktails tested in vitro (Fig. 2b). A mutant clone of Kp-P1 (KP13MC5) resistant to all existing phages was isolated from a five-phage cocktail culture and used as a target for isolating an additional phage (KP13MC5-1). The addition of this phage into cocktails extended the time until resistant bacteria appeared (~7–10 h; Fig. 2c). The same resistant clone was then used as a target to isolate seven additional phages from new sewage samples. Collectively, these eight phages targeting the resistant mutant of Kp-P1 clustered into three genomic groups and a single phage was selected to represent each group (KP13MC5-1, KP13MC5-2, and KP13MC5-5). For the final round of liquid infection dynamics testing, we generated cocktails of phages (*n* = 44) from all clusters, with 2–6 phages in each combination. A four-phage cocktail comprising phages KP13-2, KP13-16, KP13MC5-1, and KP13MC5-2 (“comb1” in Fig. 2d) successfully lysed Kp-P1 cultures at OD_600_ of 0.2 and 1.2 (representing log phase and stationary-phase bacteria, respectively) and prevented resistant mutant regrowth for at least 20 h (Fig. 2d and Supplementary Fig. 2a–d). The phage combinations used in these assays are summarized in Supplementary Data 1. Notably, the four-phage cocktail (“comb1” from Fig. 2d) also effectively suppressed the growth of cefazolin-resistant Kp-P1 cells (Supplementary Fig. 3a–c). Whole-genome sequence analyses revealed that these four selected phages belonged to four different phage genera (Webervirus, Phapecoctavirus, Slopekvirus, Teetrevirus), with genome sizes ranging from 39 kilobase pairs (kbp) to 174 kbp (Supplementary Table 3). Phage genomes were not found to carry toxic or virulent sequences (e.g., no antibiotic-resistance genes, toxins, mobile elements, or integrases were detected) when screened against relevant databases. Phage lifestyle was determined as strictly lytic based on the absence of integrase motifs in phage-predicted proteins, and on the prediction made by BACPHLIP. Finally, we confirmed the bacteriolytic ability and optimal concentration (5 × 10^9^ plaque-forming units [PFUs]/mL) by performing plaque assays (Fig. 2e).

### Oral administration of a phage cocktail reduced Kp levels in germ-free mice and SPF mice inoculated with a Kp strain derived from a patient with PSC

**Fig. 3:**
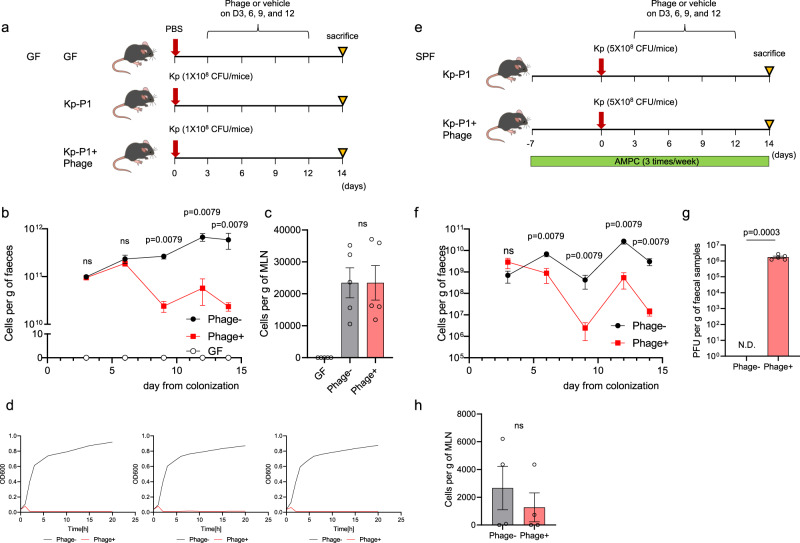
Oral administration of the phage cocktail reduced the amount of Kp in mice inoculated with a Kp strain derived from a patient with PSC. **a** Study design: GF mice inoculated with patient-derived Kp were orally administered with either the phage cocktail or the vehicle every 3 days for 2 weeks. GF mice with PBS inoculation was used as control (*n* = 5 mice per group). **b**, **c** Kp levels in fecal samples (**b**) and in MLNs (**c**). **d** Testing the inhibition of in vitro growth by a finally optimized phage cocktail against three Kp strains isolated from fecal samples of phage-treated mice. Bacterial growth was assessed via an OD_600_ of 0.2 over a 20 h period. **e** Study design: SPF mice were pretreated with AMPC 7 days before inoculation with patient-derived Kp and then orally administered with either the phage cocktail or the vehicle every three days for 2 weeks (*n* = 5 mice per group). **f** Kp levels in fecal samples. **g** Phage levels in fecal samples. N.D. not detected. **h** Kp levels in MLNs. ns not significant. The data shown represent the mean ± SEM. The two-sided Mann–Whitney test (for **b**, **f**, and **h**) or the two-sided Student’s *t* test (for **c** and **g**) was applied.

To examine the therapeutic effect of our phage cocktail targeting Kp-P1 isolated from a patient with PSC, germ-free (GF) mice were inoculated with Kp-P1 and used as the gnotobiotic mice; subsequently they were orally administered 10^9^/mL PFU of the phage cocktail described above (“comb1” from Fig. 2d) or the vehicle  every three days, and the amount of Kp was measured in both fecal samples and mesenteric lymph nodes (MLNs; Fig. 3a). The phage cocktail reduced the Kp abundances in fecal samples beginning on day 9 post-inoculation (Fig. 3b); however, the Kp levels in MLNs did not differ between the groups (Fig. 3c). We confirmed that remaining Kp strains isolated from fecal samples of phage-treated mice on day14 were not resistant to the original phage cocktail in vitro (Fig. 3d). To examine the potential effect of phage in the presence of other microbial components, as is relevant to future clinical applications, specific pathogen-free (SPF) mice pretreated with ampicillin sodium salt (AMPC) for 7 days before Kp inoculation (to promote colonization) were administered either our phage cocktail or vehicle every three days, and the Kp levels were similarly measured on day 14 (Fig. 3e). Under these experimental conditions, Kp was not detected in the liver through culture, consistent with the results obtained in gnotobiotic mice (Supplementary Fig.4). Similarly, our phage cocktail significantly reduced Kp levels (Fig. 3f) and showed abundant phage replication (Fig. 3g) in mouse fecal samples, but did not affect the Kp levels in MLNs (Fig. 3h). The suppressive effects on the fecal bacterial load lasted for a longer period, up to 4 weeks (Supplementary Fig. 5a). We also confirmed that Kp inoculation alone did not induce PSC-like hepatobiliary injuries, regardless of phage administration (Supplementary Fig. 5b–d).

### Phage-mediated alteration of the gut microbiota in Kp-colonized mice

We next sought to clarify the effect of our phage cocktail on the overall gut microbiota in Kp-colonized mice. We assigned SPF mice to two groups; those administered the phage cocktail (phage + group) and those administered the vehicle control (phage- group) (Fig. 3e).

**Fig. 4:**
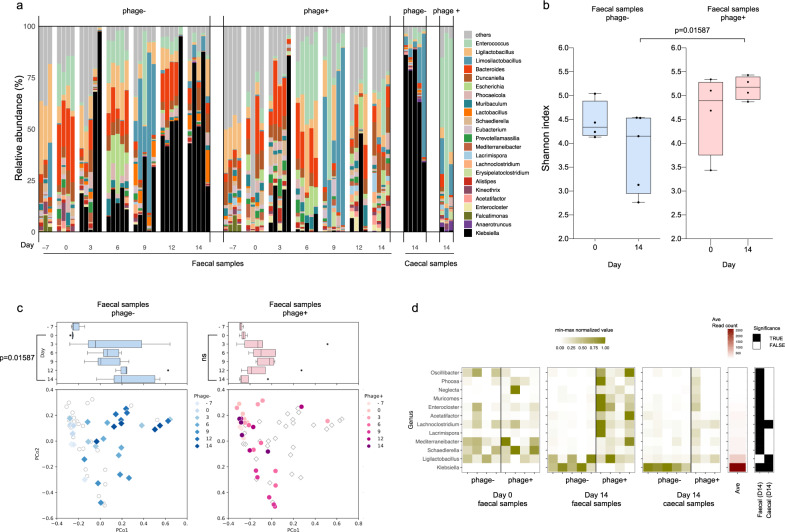
Alteration of the overall gut microbiota composition in Kp-colonized mice that were orally administered the phage cocktail. **a** Relative microbiota abundances in fecal and cecal samples collected at the indicated time points. Each genus present at ≥1% (on average) under at least one experimental condition is shown in the bar graph. *Klebsiella* spp. are represented with black bars. **b** Alpha-diversities of the microbial communities in stool from the phage −/+ groups on days 0 and 14. The alpha-diversities were calculated using the Shannon index. Each dot represents one sample. The boxes indicate the 25th to 75th percentiles, with the lines within the boxes indicating the median values. The whiskers represent the maximum and minimum values. **c** Beta-diversities of the fecal samples, demonstrating the temporal changes in the gut microbiota throughout the course of the in vivo study. Principal Coordinate Analysis (PCoA) was conducted using weighted UniFrac distances among the samples. The color density reflects the sampling time point, as indicated in the color scale. Each dot represents one sample. Boxplots showing the distributions of each time point on PCo1 are presented on top. The boxes represent the 25th to 75th percentiles, with the lines within the boxes indicating the median values. The whiskers represent the maximum and minimum values. The dots indicate outliers. **d** Normalized abundance of each genus in fecal and cecal samples on days 0 and 14. Genera with significant abundance differences between the phage −/+ groups on day 14 are shown, whereas genera that differed significantly on day 0 are excluded. The abundance of each genus was normalized by Min-Max normalization among the samples. **a**–**d** The numbers of biologically independent samples are as follows: for fecal samples, 3 phage- and 4 phage+ samples on day −7, 4 phage- and 4 phage+ samples on day 0, 5 phage− and 5 phage+ samples on days 3, 6, and 9, and 5 phage− and 4 phage+ samples on days 12 and 14. For cecal samples, there were 5 phage− and 3 phage+ samples on day 14. The two-sided Wilcoxon rank-sum exact test was applied.

The taxonomic information in each sample is summarized in Supplementary Data 2 and 3. The relative abundance of microbiota in fecal samples on day 3 showed successful Kp-P1 colonization in the gut of the mice in both groups (Fig. 4a). *Klebsiella* spp. were distinctly detected on day 3 whereas no *Klebsiella* spp. were detected before day 0. When the phage cocktail was administered intermittently, beginning on day 3, to the mice in the phage+ group, it significantly inhibited the increase in the relative abundances of *Klebsiella* spp., which remained low until day 14 (Fig. 4a, Supplementary Fig. 6a, b). In contrast, *Klebsiella* spp. dominated for the duration of the treatment in both fecal and cecal samples of mice in the phage- group. Next, we calculated the Shannon index of each fecal sample to assess the effect of the phage treatment on the alpha diversity of the gut microbiota. The Shannon indices of the phage+ samples were significantly higher than those of phage- samples on day 14 (Fig. 4b). Plausibly, *Klebsiella* spp. colonization decreased the microbial diversity, and the reduction of Kp bacterial load with phages helped preserve the alpha-diversity by triggering the growth and expansion of other gut microbes. Principal coordinate analysis (PCoA) revealed different gut microbiota-profile alterations after *Klebsiella* spp. colonization between the phage- and phage+ groups (Fig. 4c). Administering Kp-P1 induced distinct changes in the overall compositions of the microbial communities as compared to those of the original samples taken at day 0. Significantly, in mice treated with the phage cocktail, the microbiota on day 14 reverted more closely to the composition of day 0 samples. The statistical analysis showed a significant difference at day 14 compared to day 0 in the phage− groups (*p* value = 0.016), but not in the phage + group (*p* value = 0.886), suggesting that the phages enabled the preservation of the normal microbiota. To further test the specificity of the phage cocktail for *Klebsiella* spp., we focused on the genera affected by phage cocktail administration. Genera without significant differences in their abundances between the phage −/+ groups on day 0 were extracted. Among the genera, 12 genera were identified with significant differences on day 14. Of these 12 genera, except for *Klebsiella*, all were low average abundance (<6%) of 25 samples composed of day 0 fecal, day 14 fecal, and day 14 cecal samples, compared to that of *Klebsiella* (26.1%) (Supplementary Data 4). Moreover, the relative abundance of *Klebsiella* was significantly different in both fecal and cecal samples on day 14 between the phage −/+ groups (Fig. 4d). Collectively, these results suggest that our phage cocktail specifically targeted and inhibited *Klebsiella* spp. with minimal undesirable effects on the overall composition of the gut microbiota.

### Oral administration of the phage cocktail attenuated hepatobiliary inflammation and fibrosis progression

We examined whether the reduction of the Kp burden by oral phage administration might lead to the attenuation of hepatobiliary injuries. A single Kp inoculation immediately before initiating 3, 5-diethoxycarbonyl-1, 4-dihydrocollidine (DDC) feeding exacerbated the course of hepatobiliary inflammation and fibrosis in the livers of SPF mice when inflammatory immune responses were assessed on day 21 (Supplementary Fig. 7a–i), supporting the pathogenic role of patient-derived Kp in the development of hepatobiliary injuries. AMPC administration was required during the entire experimental duration for sufficient Kp colonization and translocation to the MLNs under SPF conditions, consistent with the findings of a previous report^19^. The bioinformatics approach revealed that Kp-P1 does not have unique characteristics in terms of the genome size, GC (%) content, overall number of genes, number of AMR genes, and number of virulence genes compared with other Kp strains from PSC patients in an international cohort with available genome data (Supplementary Table 4).

**Fig. 5:**
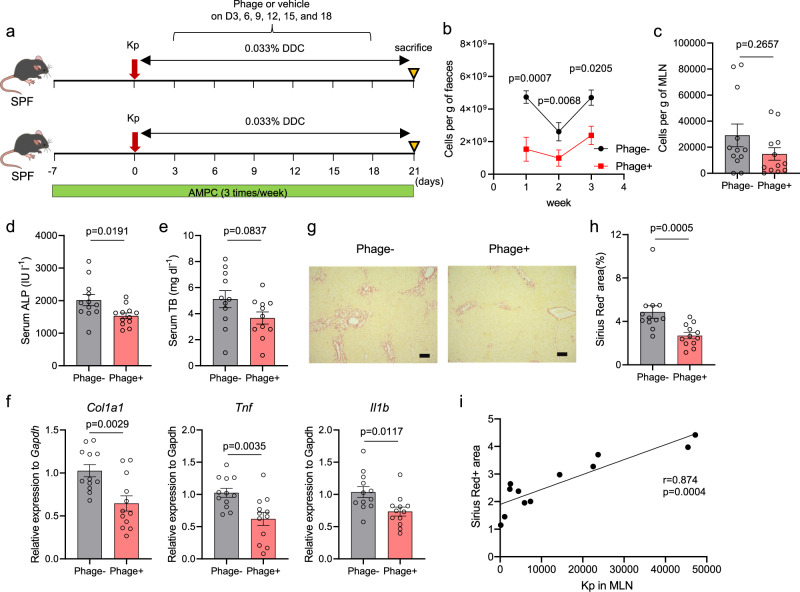
Oral administration of the phage cocktail attenuated DDC-induced hepatobiliary inflammation and fibrosis progression. **a** Study design: SPF mice were pretreated with AMPC 7 days before being inoculated with patient-derived Kp and then orally administered the phage cocktail or the vehicle every three days for 3 weeks during DDC feeding (*n* = 12 mice per group). **b**, **c** Amount of Kp in fecal samples (**b**) and MLNs (**c**), as assessed using qPCR. **d**, **e** Serum ALP levels (**d**) and TB levels (**e**) of the mice. **f** qPCR analysis of *Col1a1, Tnf*, and *Il1b* mRNA expression relative to *Gapdh* mRNA expression in whole mouse livers. **g** Representative photomicrographs of Sirius red-stained liver sections from the mice. Scale bars, 100 μm. **h** Quantitation of the Sirius red-positive areas of the mouse liver sections. The data shown represent the mean ± SEM. The two-sided Mann–Whitney test (for **b,** **c**, and **h**) or the two-sided Student’s *t* test (for **d**–**f**) was applied. Data are combined from several independent experiments. **i** Correlation between the amount of Kp in MLNs and the degree of liver fibrosis, assessed via quantitation of the Sirius red-positive areas of the liver sections in the phage-treated mice (*n* = 12 mice). Spearman’s rank correlation test was applied.

To examine the effect of our phage cocktail on hepatobiliary injury, Kp-colonized mice were orally administered the phage cocktail or vehicle  every three days during DDC feeding for 3 weeks, followed by evaluation on day 21 (Fig. 5a). Consistently, we observed a downward trend in fecal Kp levels starting from day 7 (Fig. 5b), whereas a significant reduction in Kp levels in the MLNs was not achieved through phage treatment (Fig. 5c). Serum levels of ALP and TB, and hepatic expression levels of inflammation-related genes were significantly lower in phage-treated mice (Fig. 5d–f). Furthermore, phage-treated mice showed significantly less liver fibrosis than untreated mice, as evaluated using liver histology (Fig. 5g, h) and fibrosis-related gene expression (Fig. 5f). These results confirmed that intestinal Kp colonization was reduced by the phage cocktail but translocation to the MLNs was not completely prevented, leading to a partial attenuation of liver injuries. Notably, the amount of Kp in the MLNs was significantly correlated with the degree of fibrosis in the phage-treated mice (Fig. 5i), reinforcing that direct targeting of the translocated Kp may be a more appropriate therapeutic approach.

Regarding the comprehensive effect of the phage cocktail against Kp, we confirmed that the suppressive effect was strain-specific, as the phage cocktail did not suppress the growth of another Kp strain isolated from a patient with PSC in our cohort, Kp-P5 in vitro (Supplementary Fig 8a and Supplementary Table 4) and failed to ameliorate hepatobiliary injuries in SPF mice colonized with this Kp strain (Supplementary Fig 8b–h).

### Intravenous administration of the phage cocktail attenuated hepatobiliary inflammation and fibrosis progression

**Fig. 6:**
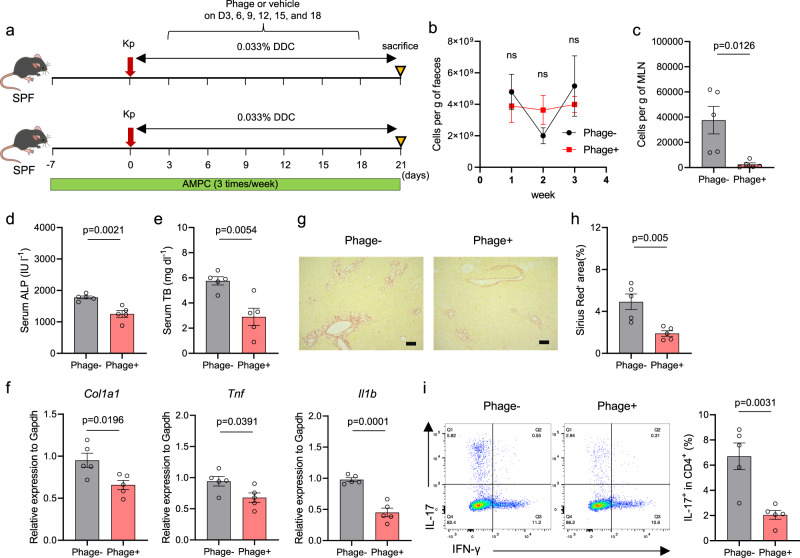
Intravenous administration of the phage cocktail attenuated MLN bacterial levels and DDC-induced hepatobiliary inflammation and fibrosis progression. **a** Study design: SPF mice were pretreated with AMPC 7 days before being inoculated with patient-derived Kp and then intravenously administered with the phage cocktail or the vehicle every three days during DDC feeding for 3 weeks (*n* = 5 mice per group). **b**, **c** Kp levels in fecal samples (**b**) and MLNs (**c**) as assessed by qPCR. **d**, **e** Serum ALP levels (**d**) and TB levels (**e**) of the mice. **f** qPCR analysis of *Col1a1, Tnf*, and *Il1b* mRNA expression relative to *Gapdh* mRNA expression in whole mouse livers. **g** Representative photomicrographs of Sirius red-stained liver sections from the mice. Scale bars, 100 μm. **h** Quantitation of the Sirius red-positive areas of the mouse liver sections. **i** Representative flow cytometric staining of intracellular IL-17 and IFN-γ in the liver (left) and the frequency of IL-17^+^ CD4^+^ T cells (right). The data shown represent the mean ± SEM. The two-sided Mann–Whitney test (for **b**) or the two-sided Student’s *t* test (for **c**–**f**, **h**, and **i**) was applied. ns not significant.

Given that intravenous (IV) administration of phages achieved a superior effect on the delivery of phages to the MLN compared to the oral approach (Supplementary Fig. 9a, b), we finally examined the effect of IV administration of the phage cocktail on Kp-induced hepatobiliary injuries. The phage cocktail was administered intravenously for 3 weeks (every three days) during DDC feeding, consistent with the protocol used for oral administration (Fig. 6a). In contrast to the results found after oral administration, IV administration had little effect on the fecal Kp levels (Fig. 6b), but significantly reduced the translocated Kp levels in the MLNs (Fig. 6c). Consequently, mice treated intravenously with the phage cocktail showed significantly attenuated DDC-induced hepatobiliary inflammation and fibrosis progression, based on serology, histology, and gene expression results in the liver (Fig. 6d–h). Notably, Th17 cell induction (but not Th1 cell induction) in the liver was also significantly attenuated by IV phage administration (Fig. 6i), reinforcing the role of translocated Kp in the pathogenesis of hepatobiliary injuries in this model.

## Discussion

Recent studies on the microbiome have indicated an association between dysbiosis and PSC; however, the clinical implications and significance of disease-specific microbiota as a potential therapeutic target of PSC remain elusive. In this study, we identified a clear correlation between the presence of both Kp and Eg with disease progression by analyzing the long-term clinical courses of patients. Furthermore, the clinical relevance of a single Kp pathobiont was confirmed by demonstrating the therapeutic effect of bacteriophages that target Kp in murine hepatobiliary injury models.

As >70% of PSC cases are complicated by UC^1,2^, colonic inflammation is a plausible cause of the barrier dysfunction; however, most patients with UC do not have PSC^20,21^, suggesting an additional layer of complexity. It is widely known that PSC-associated UC (PSC/UC) represents a distinct disease phenotype. PSC/UC typically exhibits discontinuous right-sided colitis with backwash ileitis, whereas classical UC is characterized by continuous lesions originating from the rectum^21–23^. Here, we demonstrated that the disease-associated bacteria Kp and Eg, which were initially found to be prevalent in patients with PSC/UC, were also enriched in PSC patients without UC. Notably, the prevalence was not affected by the dominance of intestinal lesions in patients with PSC/UC. These results collectively highlight the universal role of these pathobionts in PSC, regardless of the presence of intestinal lesions. It is tempting to speculate whether the prevalence of a single pathobiont or the combination of two pathobionts affects the clinical course of PSC, as demonstrated in gnotobiotic mouse models. Notably, individuals carrying the Kp + Eg combination displayed elevated serum ALP levels and experienced poorer clinical outcomes. As a limitation of the current findings, it remains unclear whether these patients carried these specific components of microbiota at the time of PSC/UC diagnosis. For clarity, large-scale, long-term, prospective observational studies are required. Nonetheless, our findings suggest that specific microbes may serve as biomarkers and therapeutic targets in PSC. Among them, we narrowed the therapeutic target to Kp based on the pathogenicity of intestinal damage, bacterial translocation, hepatic Th17 response, and susceptibility to hepatobiliary injuries in gnotobiotic mice, which have not been demonstrated by two other pathobionts, Pm and Eg, as indicated in our previous study.

The causative role of the gut microbiota in PSC pathogenesis is supported by interventional studies targeting or manipulating the gut microbiome. A handful of small randomized clinical trials and case series have shown short-term biochemical improvements with antibiotic treatments (primarily vancomycin and metronidazole) in patients with PSC^8^; however, the emergence of multidrug-resistant strains, including Kp strains categorized as carbapenem-resistant *Enterobacteriaceae*, has hampered their long-term use. To overcome these limitations, we generated a bacteriophage cocktail specifically targeting a Kp strain (derived from a patient with PSC) and successfully demonstrated its efficacy in a murine model of hepatobiliary injury. The advantage of phage therapy over antibiotic therapy is generally attributed to its safety and strain specificity. Regarding safety, we confirmed that the administered phages had little effect on murine hepatobiliary injuries, both in serological and histological terms. In addition, our group has recently demonstrated the safety and persistent accumulation of orally administered Kp phages in the lower gut of healthy human volunteers^18^. Detailed longitudinal bacterial analysis using 16 S rRNA gene sequencing revealed that the phage cocktail developed in this study abolished the colonization of Kp without inducing off-target dysbiosis, which is consistent with previous literature^13^. Furthermore, we confirmed the suppressive effect on an engineered antibiotic-resistant Kp strain in vitro, supporting the superior and additional therapeutic effect of phage beyond antibiotics, as demonstrated in other microbiome-related human diseases^15,16^.

Oral administration of our phage cocktail significantly reduced the Kp levels in fecal samples and subsequently attenuated hepatobiliary injuries and liver fibrosis exacerbated by inoculation with Kp. Successful disease attenuation in the liver following oral administration of phage targeting specific gut bacteria has also been reported in murine models of alcoholic liver disease^24^ and non-alcoholic liver disease^25^. Given that bacterial translocation plays a substantial role in the pathogenesis of several intractable extraintestinal diseases including PSC^6^ and autoimmune hepatitis^26,27^, it may be beneficial to directly target translocated pathogenic bacteria. In this regard, we demonstrated that the intravenous administration of phages can successfully deliver phages and reduce the bacterial burden in MLN and subsequent Th17 immune responses in the liver, with little effect on the fecal content. However, it should be noted that long-term systemic phage administration may induce immunogenicity in the host^28^. Considering its non-inferior therapeutic effect on the hepatobiliary injuries compared to that achieved by oral administration, further validation may be needed to determine the appropriate method (such as the dose, timing, duration, and route of administration) for phage administration in future clinical applications for PSC.

This study demonstrated a clear association between the specific gut microbiota, Kp, and the clinical course of PSC, which aligns with the results of a recent report by a research group from Norway^29^. Targeting a single Kp pathobiont using a strain-specific phage cocktail holds promise as a potential approach for treating PSC. However, it is worth noting that this approach may necessitate the use of individual strain-specific phages. There are a few limitations to consider in this study. First, the results obtained from the clinical information rely on a small number of patients; thus, further validation with a larger sample size is necessary. Second, although the phage treatment resulted in an approximate 2-log reduction in the abundance of Kp, a significant number of pathogens still persisted in the body. Therefore, it is crucial to ascertain whether further suppression of bacterial counts, ideally leading to complete elimination, is necessary to ameliorate bile duct damage, particularly in the long-term analysis model, given the chronic nature of this disease. Lastly, our study focused on targeting Kp as a potential causative bacterial pathogen of PSC. However, it is important to acknowledge that targeting multiple potential pathobionts of PSC, as demonstrated in another study^30^, may yield synergistic effects. While such an approach can help verify the feasibility of phage treatment for PSC in humans, the development of a pan-Kp phage cocktail, capable of covering the majority of pathogenic Kp strains (which is currently being developed by our team), may pave the way for broader clinical applications in the treatment of PSC.

## Methods

### Patients

Forty-five patients with PSC complicated with IBD (*n* = 34) or without IBD (*n* = 11) were included in this study. PSC was diagnosed according to clinical guidelines and typical cholangiography findings (endoscopic retrograde cholangiography and/or magnetic resonance cholangiopancreatography) or liver biopsy. All patients underwent at least one colonoscopy to exclude the presence of IBD. The IBD subtype was classified according to standard disease descriptions based on a combination of endoscopic, histopathological, radiological, and serological investigations^31,32^. IBD unclassified was diagnosed by combining the features of UC with those of ileal disease compatible with Crohn’s disease. The ethics committee at Keio University School of Medicine approved the study protocol (approval #20140211). The study was conducted in accordance with the principles of Helsinki Declaration II, and written informed consent was obtained from all study participants. In the case of minors, written informed consent was obtained from their parents. No compensation was provided to research participants. The study was registered in the University Hospital Medical Information Network (UMIN) clinical trial registration system (UMIN 000018068).

### Animals

Male GF mice (C57BL/6 background strain, 6–8-weeks old) were purchased from Sankyo Lab Service Corporation (Tokyo, Japan) and kept in the GF Facility of Keio University School of Medicine. Male C57BL/6 mice (6–8-weeks old) were purchased from Japan CLEA (Tokyo, Japan) and maintained under SPF conditions. The mice were kept in the Central Laboratories for Experimental Animals (Kawasaki, Japan) and the Animal Care Facility of Keio University School of Medicine, with a 12-hour light/dark cycle, a temperature range of 22–25 °C, and a relative humidity of 45–55%. The animal experiments conducted between September 2019 and February 2023 were approved by the animal ethics committee of Keio University and were performed in accordance with institutional guidelines and home office regulations.

### Bacterial strains and culture

PSC-derived Kp strains (Kp-P1 and Kp-P5) isolated from the MLNs of GF mice transplanted with human stool from patients with PSC at Keio University were used. Cefazolin-resistant Kp-P1 was produced by culturing Kp-P1 in 200 μL of brain heart infusion broth (BHI broth) (Becton Dickinson) containing cefazolin (128, 64, 32, 16, 8, 4, 2, 1 μg/mL) in a 96-well plate at 37 °C for 24–48 h. Bacterial species were preserved in BHI broth containing 40% glycerol, snap-frozen, and stored at −80 °C until use. The frozen stock was thawed, applied to deoxycholate-hydrogen sulfide-lactose agar (Nissui), and incubated at 37 °C overnight. The colonies were cultured in 10 mL of BHI broth at 37 °C for 15–20 h. To prepare the bacteria, they were collected by centrifugation (2000 × *g*, 10 min, 4 °C) and dissolved in phosphate-buffered saline (PBS).

### Phage administration

Either the bacteriophage cocktail or vehicle control (phage buffer) was administered orally or intravenously. Phage buffer is composed of 50 mM Tris-HCl, 100 mM NaCl, 5 mM MgCl_2_, and 0.1 mM MnCl_2_ pH=7.5 in DDW. For oral administration, either the bacteriophage cocktail (1 × 10^9^ PFUs) or vehicle control suspended in 200 μL of medium (phage buffer with 2.6% of NaHCO_3_) was administered to mice every 3 days. For intravenous administration, either the bacteriophage cocktail (1 × 10^8^ PFUs) or vehicle control suspended in 100 μL of medium (phage buffer) was administered to mice every 3 days.

### Gnotobiotic study design

To examine the effects of the bacterial species, 1 × 10^8^ colony-forming units (CFUs) of a single species suspended in 200 µL of medium were orally administered using a stainless-steel feeding needle to GF mice. The experiments were conducted after a colonization period of 2 weeks. During this period, either the bacteriophage cocktail or vehicle control was orally administered to mice every 3 days, and fecal samples were collected on days 3, 6, 9, and 12 before bacteriophage administration. The number of bacteria in each sample was assessed by counting the CFUs in each homogenized sample per unit weight.

### SPF mouse study design

SPF mice were pretreated with a single dose of AMPC (6.7 g/dL, 200 μL; Sigma-Aldrich) 7 days before inoculation with Kp (5 × 10^8^ CFUs) to promote colonization. The experiments were conducted after a colonization period of 2–4 weeks. After colonization, either the bacteriophage cocktail or vehicle control was orally or intravenously administered to mice every 3 days, and fecal samples were collected immediately before bacteriophage administration. AMPC was administered continuously every 3 days during the experiment. The number of bacteria in each sample was evaluated using quantitative polymerase chain reaction (qPCR) analysis in each homogenized sample per unit weight.

### DDC-induced experimental hepatobiliary inflammation and liver fibrosis

The colonization method was the same as that used in the SPF mouse study design. To induce hepatobiliary inflammation and liver fibrosis, SPF mice were freely fed a 0.033% DDC (Sigma-Aldrich, Tokyo, Japan)-enriched diet just after colonization for 21 days, followed by serological, histological, and immunological assessments. To examine the effect of bacteriophages, Kp-colonized SPF mice were orally or intravenously administered the phage cocktail or vehicle three times per week during DDC feeding for 3 weeks.

### Sample collection and DNA extraction

Mouse fecal and cecal samples were collected and stored at −80 °C until the following processes were performed. The samples were suspended in 800 μL of TE10 buffer (10 mM Tris-HCl, 10 mM EDTA, pH 8.0) and incubated at 37 °C for 1 h after adding 100 μL of TE10 buffer containing lysozyme from chicken egg whites (final concentration of 14–17 mg/mL, Sigma-Aldrich). Purified achromopeptidase (final concentration of 1904–2174 U/mL, FUJIFILM Wako Pure Chemical or Sigma-Aldrich) was added to each fecal or cecal suspension, and each suspension was further incubated at 37°C for 30 min. Proteinase K (final concentration of 0.8–1 mg/mL, Merck) and sodium dodecyl sulfate (final concentration of 0.8–1%, FUJIFILM Wako Pure Chemical) were added, and the mixtures were incubated at 55 °C for 1 h. Fecal and cecal DNA was extracted with phenol: chloroform: isoamyl alcohol (25:24:1, Thermo Fisher Scientific), and 800 μL of the aqueous top layer was retrieved. The extracted DNA was precipitated by adding 80 μL of 3 M sodium acetate buffer (pH 5.2 ± 0.1, Sigma-Aldrich) and 800 μL of cold isopropanol (Nacalai Tesque) to the top layer and washed once in 75% ethanol (Nacalai Tesque) solution. The precipitated DNA extract was dissolved in 100 μL of TE buffer (10 mM Tris-HCl, 1 mM EDTA, pH 8.0) with RNase A (final concentration of 100 μg/mL, FUJIFILM Wako Pure Chemical) and then incubated at 37 °C for more than 1 h. The DNA was precipitated by adding an equal volume of cold 20% polyethylene glycol (molecular weight: 6000 g/mol, Cosmo Bio) solution in 2.5 M sodium chloride (Cosmo Bio) and washed twice in a 75% ethanol solution. The purified DNA was dissolved in TE buffer.

### Quantification of fecal microbiota using qPCR

Quantification of Kp was performed using a *Klebsiella pneumonia-*EASY Genesig Kit (Primerdesign Ltd., UK), PrecisionPLUS qPCR Master Mix (Primerdesign Ltd.), and the Thermal Cycler Dice Real-Time System II (Takara), according to the manufacturer’s instructions. Quantification of Eg and Pm was performed using SYBR Premix EX Taq II (Takara) and the Thermal Cycler Dice Real-Time System II based on a standard curve generated from a positive control produced by DNA extraction of 1×10^8^ CFUs of single strain. The primer sets used in this study were as follows: Eg, forward 5′-TTACTTGCTGATTTTGATTCG-3′ and reverse 5′-TGAATTCTTCTTTGAAATCAG-3′^33^; Pm forward 5′-GTTATTCGTGATGGTATGGG-3′ and reverse 5′-ATAAAGGTGGTTACGCCAGA-3′.

### 16S rRNA gene sequencing and analysis

The hypervariable V3-V4 region of the 16S rRNA gene was amplified by PCR using the 341Fmod (5′-CCTACGGGNGGCWGCAG-3′) and 805R (5′-GACTACHVGGGTATCTAATCC-3′) primers. The PCR products were purified using AMPure XP magnetic beads (Beckman Coulter), and the DNA concentrations were measured using a Quant-iT Picogreen dsDNA Assay Kit (Thermo Fisher Scientific) and an Infinite M Plex plate reader (Tecan). The pooled amplicon libraries were sequenced using the Illumina MiSeq platform and MiSeq Reagent Kit v3 (600 cycles; paired-end 2 × 300 bp), according to the manufacturer’s instructions. For subsequent analyses, the sequenced reads were de-multiplexed and their PCR primer sequences were trimmed using Cutadapt (v3.3).

For the microbial-compositional analysis, amplicon sequence variants (ASVs) were constructed using the DADA2 pipeline^34^ (v1.18.0) with the maxN = 0, truncQ = 2, and truncLen = c(240,220) parameters for the filterAndTrim function. After removing the chimeric reads, 10,000 merged reads were randomly extracted for each sample and analyzed further. We avoided the use of the samples with <10,000 merged reads to prevent biases derived from variations in the number of sequencing reads. Microbial alpha- and beta-diversities were estimated using the Shannon index and unique fraction (UniFrac) distance measures^35^, respectively (Scikit-bio; v0.5.5.post13-11). PCoA was performed using weighted or unweighted UniFrac distances. Taxonomic assignments for each ASV were conducted using the GLSEARCH program^36^ with the 16S RefSeq database^37^ downloaded from the National Center for Biotechnology Information on August 19, 2021. The relative ASV abundances at the genus level were summarized from the taxonomic assignment results, using 94 % sequence identity as the threshold. For a comparative analysis between the results obtained with different experimental conditions, Wilcoxon rank-sum exact test (coin; v1.4.2) was conducted to study the alpha-diversity, beta-diversity, and the relative abundance of each genus present at ≥0.1% on average under at least one experimental condition.

### Lymphocyte isolation and flow cytometry

Liver and spleen mononuclear cells (MCs) were isolated according to a previously described method^38^. Briefly, livers were perfused with PBS through the portal vein, minced, and then passed through a 100 μm nylon mesh. The resulting filtrate was centrifuged at 50 × *g* for 1 min, and the supernatant was subsequently washed once. The cells were then suspended in a Histopaque solution (Sigma-Aldrich) and layered over an HBSS solution. Following centrifugation at 2500 rpm for 20 min, the cells were collected from the upper surface of the Histopaque solution. After blocking with anti-FcR (CD16/32, BD Pharmingen) for 20 min, the cells were incubated with a specific fluorescence-labeled monoclonal antibody diluted 100-fold at 4 °C for 30 min. The following monoclonal antibodies were used: anti-mouse anti-TCRβ (catalog number 109228, BioLegend, PerCP/Cy5.5 conjugate, clone H57-597), anti-CD3e (catalog number 552774, BD Bioscience, PE-cy7 conjugate, clone 145-2C11), anti-CD4 (catalog number 563106, BD Bioscience, BV510 conjugate, clone RM4-5), and fixable viability dye eFluor780 (catalog number 65-0864-14, eBioscience). Events acquired with FACS Canto II flow cytometer (Becton Dickinson) were collected using FACSDiva and analyzed using FlowJo software (Tree Star Inc.). For intracellular cytokine staining, cells were stimulated for 4 h with phorbol-12-myristate-13-acetate (50 ng/mL; Sigma-Aldrich) and ionomycin (500 ng/mL; Sigma-Aldrich) in the presence of brefeldin A (10 µg/mL; BD bioscience) or Golgistop (10 µg/mL; BD Bioscience), followed by surface staining, permeabilization, and intracellular staining with anti-mouse anti-IFN-γ (catalog number 554412, BD Bioscience, PE conjugate, clone XMG1.2), anti-IL-17A (catalog number 560221, BD Bioscience, Alexa Fluor488 conjugate, clone TC11-18H10), anti-IL-22 (catalog number 17-7222-82, eBioscience, APC conjugate, clone IL22JOP), and anti-RORγt (catalog number 562894, BD Bioscience, BV421 conjugate, clone Q31-378) diluted 30-fold at 4 °C for 30 min.

### Serological and histological analyses

Serum alanine transaminase (ALT), alkaline phosphatase (ALP) and total bilirubin (TB) levels were measured using DRI-CHEM (Fuji Film, Tokyo, Japan) following the manufacturer’s instructions. Following removal, livers were fixed in 10% formalin and embedded in paraffin. Histological analysis was performed with hematoxylin and eosin (H&E), Masson’s trichrome, and Sirius red staining using paraffin-embedded liver sections. Samples were observed under the BZ X-700 fluorescence microscope (Keyence). The degree of fibrosis was assessed by measuring the positively stained area with Sirius red using ImageJ software (NIH, Maryland, USA).

### qPCR analysis

Total RNA was extracted from the liver and spleen using an RNeasy Mini Kit (Qiagen, Venlo, Netherlands), and complementary DNA (cDNA) was synthesized from 1 µg total RNA using the iScript cDNA Synthesis Kit according to the manufacturer’s instructions (Bio-Rad, Hercules, CA, USA). Gene expression analysis was performed using SYBR Green or TaqMan PCR assays (Thermo Fisher Scientific) with a Thermal Cycler Dice Real-Time System (Takara Bio). Target gene expression was normalized to that of *Gapdh*. Primer information is summarized in Supplementary Table 5.

### Liquid infection dynamics - Time to mutant (TTM)

Bacterial strains were inoculated from glycerol stocks stored at −80 °C, streaked on BHIS agar plates (Novamed, Israel), and incubated overnight at 37 °C. Several colonies of each bacterial strain were picked into 4 mL of liquid BHIS (BD BACTO^TM^ Brain Heart Infusion BHI medium supplemented with 0.5% Yeast Extract). The strains were grown at 37 °C with agitation to OD_600_ = 1.5 and diluted 1:1000 in fresh BHIS medium. They were then cultured again at 37 ˚C with agitation until the OD_600_ reached the desirable values of 0.2 and 1.2 at which time MMC ions (final concentration of 1 mM each of CaCl_2_, MgCl_2_ and MnCl_2_) were added to the culture and 200 µL was dispensed per well in a 96-well plate. Each phage was diluted from the phage stock to 10^8^ PFU/mL and combined into equimolar cocktails, from which 10 µL of each cocktail (10^6^ total PFU) was added to the designated wells, in triplicates. BHIS media served as blank, and bacteria without phages added served as no phage control. Mineral oil (Thermo Scientific Acros #415080010) was added at 50 µL to each well to limit evaporation of the sample, followed by covering with a thin sterile optically transparent film (Sigma #Z369667) to keep the culture sterile. Plates were incubated overnight in a plate reader at 37 °C with agitation, and OD_600_ was measured every 15 min.

### Drop/spot plaque assay

Multiple colonies of each bacterial strain were selected and transferred into 4 mL of liquid BHIS medium. The strains were then incubated at 37 °C and 180 rpm until the OD600 reached approximately 1.5. Next, 150 µL of each culture was combined with 4 mL of molten top agar (BACTOTM Brain Heart Infusion medium containing 0.4% Agarose) supplemented with MMC and poured onto a BHIS agar plate. Following a 30-min incubation at 37 °C, 5 µL of the sample to be tested was added. Plaque assays were conducted using 10-fold serial dilutions of filtered enrichments. The plates were then incubated overnight at 37 °C.

### Assessment of phage counts in fecal samples and MLNs

Fecal samples were weighed and mashed in a sterile dish with 500 µL of sterile PBS. The sample was transferred into a new tube and centrifuged for 4500 × *g* for 10 min at 4 °C. The supernatant was filtered through 0.22 µm sterile filters (Merck) and serially diluted in PBS. Next, 5 µL of each diluted solution was spotted onto BHIS plates covered with 0.4% agar overlay BHIS, inoculated with the relevant bacterial host strain and supplemented by 1 mM of MMC ions. The plate was dried and incubated at 37°C, until the appearance of phage plaques. Phage counts were determined by counting the plaques visible on the bacterial lawn. If no plaque was visible, 100 µL of the undiluted solution was spotted on the plate. If no plaque was also visible regardless of the attempt, we determined that no plaque was detected.

MLNs were weighed and homogenized using BioMasher II (Nippi) with 100 µL of sterile PBS. The sample was mixed with 400 µL of sterile PBS and centrifuged at 4500 ×*g* for 10 min at 4 °C. The supernatant was filtered through 0.22 µm sterile filters (Merck). Thereafter, 100 µL of the undiluted solution was spotted on the plate and phage counts were determined, as described previously. If no plaque was visible, we determined that no plaque was detected.

### Phage isolation

Plaques having different morphologies were picked up using a needle loop into 100 µL phage buffer (Tris-HCl pH 7.5 50 mM, NaCl 100 mM, MgCl_2_*6H_2_O 5 mM, MnCl_2_*4H_2_O 0.1 mM in DDW) and re-isolated for a total of three isolation rounds using the spot/drop plaque assay.

### Phage amplification

Several colonies of each bacterial strain were picked up and added to 4 mL of liquid BHIS. The strains were incubated at 37 °C, 180 rpm until OD_600_ was approximately 1.5. Cultures were diluted 1:1000 with BHIS medium in 5 mL and incubated again at 37 ˚C in the shaker incubator. When an OD_600_ of 0.1 was reached, 1 mM of MMC ions was added, and one plaque from the third isolation was picked up, using a needle loop, and added to the culture. Cultures were incubated at 37 °C with the agitation of 130 rpm, overnight. On the day after, cultures were centrifuged at 4 °C, 4500 × *g* for 10 min. The supernatant was filtered with a 0.45 µm syringe filter.

### Phage titer determination

To determine the titer of stock cultures of each of the phage, a drop/spot plaque assay was performed using serial 10-fold dilutions of the phage isolates. The plate was incubated overnight before counting plaques (in the dilution where 10–50 plaques could be counted) after which the titer was determined by multiplying the number counted by 200 and the reciprocal of counted dilution and expressed as plaque forming units per milliliter (PFU/mL).

### Phage DNA extraction and sequencing

Phage DNA was extracted from 0.2 mL of phage at least 1×10^8^ PFU/mL using Thermo Scientific GeneJet Genomic DNA Purification Kit (Catalog #K0721) and the manufacturer’s protocol. After extraction, the DNA concentration was determined via Qubit and Illumina libraries were created following the protocol of and sequenced using paired-end reads with an average coverage of ~250 reads per position.

### Phage bioinformatic analyses

Phage sequencing reads were assembled using SPAdes 3.10.1^39^. To finalize the genomes, assemblies were analyzed using PhageTerm 1.0.12^40^. Open reading frame prediction was performed using PATRIC^41,42^. The taxonomy of the phages was assigned based on close genomic relatedness to known phages with assigned taxonomies published by the ICTV^43^, using the recommended thresholds (>95% for species demarcation and over 70% for genus demarcation). The screening was performed for undesirable genes in the phage genomes, using BLAST to compare phage genes, against a manually created database of toxic genes based on US CFR, title 40, section 725.421 (i.e., protein synthesis inhibitor, neurotoxins, oxygen labile cytolysins, toxins affecting membrane function, molecules affecting membrane integrity and cytotoxins). The presence of antibiotic-resistance genes was analyzed using the NCBI AMRfinder tool^44^ with default parameters (version 3.6.15 with database version 2020-03-20.1). Other toxins or virulence genes were analyzed based on the PATRIC annotation pipeline. Lysogeny potential was tested by indentifying phage integrases based on the latest Hidden Markov Models (HMM) in PFAM (version 33.1)^45^ and PANTHER^46^ databases. Integrase domains were searched using HMMER version 3.3^47^. Furthermore, PHACTS v0.3^48^ and the more updated BACPHLIP v0.9.3^49^ were used to assess the potential of a virulent-only lifestyle of the phages.

### Statistical analysis

Statistical analyses were performed using GraphPad Prism software (version 8; GraphPad Software Inc.). Differences between the two groups were evaluated using the two-sided Student’s *t* test, Mann–Whitney test, and Wilcoxon rank-sum test as appropriate. Comparison of more than two groups was performed using one-way analysis of variance (ANOVA), followed by Tukey’s multiple-comparison test. Correlations were tested for significance using the Spearman’s rank correlation test. The proportions between the two groups were compared using Fisher’s exact test or the Chi-square test. Kaplan–Meier analysis was used to determine cumulative survival percentages, and differences among the groups were compared using the log-rank tests. Differences were considered statistically significant at *P* < 0.05.

### Reporting summary

Further information on research design is available in the Nature Portfolio Reporting Summary linked to this article.

## Supplementary information

Supplementary Information

Description of Additional Supplementary Files

Dataset 1

Dataset 2

Dataset 3

Dataset 4

Reporting summary

## Data Availability

16S rRNA sequencing and whole-genome sequencing data have been deposited in the DDBJ database with accession numbers DRA015605 for 16S rRNA sequencing, PRJNA887913 for the whole-genome sequencing of *Klebsiella* phages, and PRJDB7545 for the whole-genome sequencing of *Klebsiella pneumoniae* strains used in this study. Source data are provided with this paper.

## Source data

Source Data
