# Supplementary material for: Bacteriophage therapy against pathological Klebsiella pneumoniae ameliorates the course of primary sclerosing cholangitis
Source: Nat Commun. 2023 Jun 5;14:3261. doi: 10.1038/s41467-023-39029-9 (PMC10241881; doi:10.1038/s41467-023-39029-9)
Supplement: Supplementary file 1 — Supplementary Information [file 41467_2023_39029_MOESM1_ESM.pdf]

Supplementary Information

Bacteriophage therapy against pathological *Klebsiella pneumoniae* ameliorates the course of primary sclerosing cholangitis

Masataka Ichikawa,[1] Nobuhiro Nakamoto,[1*] Sharon Kredo-Russo,[2] Eyal Weinstock, [2] Iddo Nadav Weiner,[2] Efrat Khabra, [2] Noa Ben-Ishai, [2] Dana Inbar, [2] Noga Kowalsman, [2] Ron Mordoch, [2] Julian Nicenboim, [2] Myriam Golembo, [2] Naomi Zak, [2] Jagoda Jablonska, [2] Hila Sberro-Livnat, [2] Sharon Navok, [2] Nufar Buchshtab,[2] Takahiro Suzuki,[1] Kentaro Miyamoto,[1] Toshiaki Teratani,[1] Sota Fujimori,[1] Yoshimasa Aoto,[3] Mikiko Konda,[3] Naoki Hayashi,[3] Po-Sung Chu,[1] Nobuhito Taniki,[1] Rei Morikawa,[1] Ryosuke Kasuga,[1] Takaya Tabuchi,[1] Shinya Sugimoto,[1] Yohei Mikami,[1] Atsushi Shiota, [4,5] Merav Bassan, [2] Takanori Kanai[1, 6*]

[1]Division of Gastroenterology and Hepatology, Department of Internal Medicine, Keio University
[2]BiomX Ltd., Israel
[3]JSR-Keio University Medical and Chemical Innovation Center (JKiC), JSR Corp., Tokyo, Japan
[4]Department of Microbiology and Immunology, Keio University
[5]Microbiopharm Japan, Co. Ltd., Tokyo, Japan
[6]Japan Agency for Medical Research and Development, AMED, Tokyo, Japan

Inventory of Supporting Information

Supplementary Figures 1 to 9
Supplementary Tables 1 to 5

Supplementary Figure 1

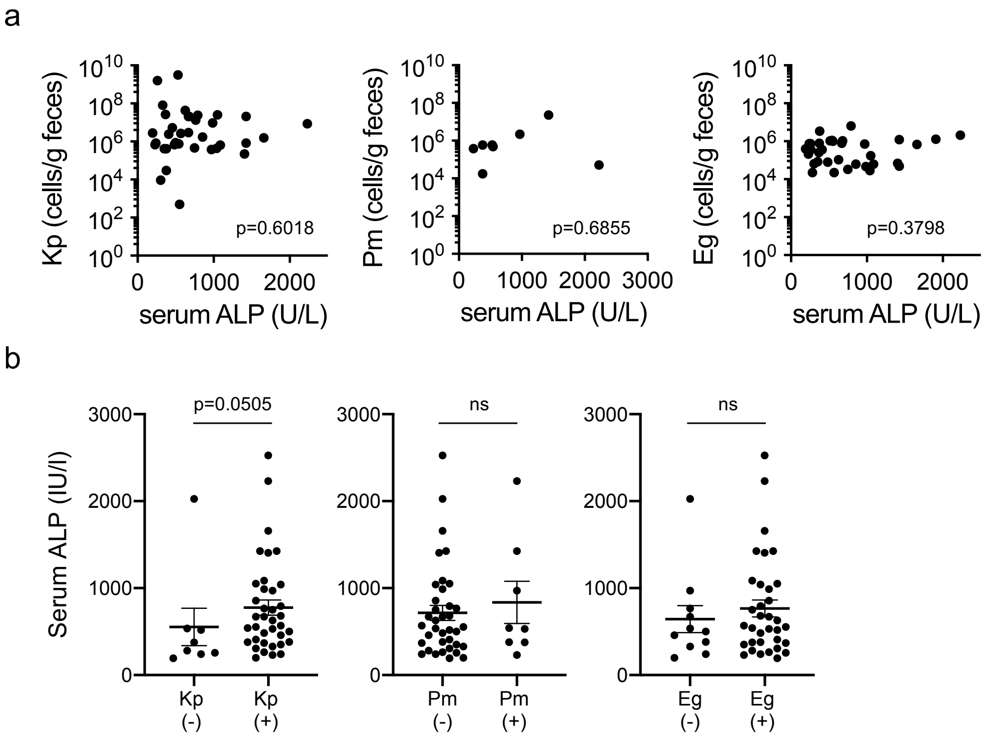

Supplementary Figure 1. The abundance of each bacterium did not affect the serum hepatobiliary enzyme levels. a The correlation between the amount of each bacterium and the serum ALP levels (n = 45 patients). The Spearman's Rank Correlation Coefficient was applied. **b** Serum ALP levels classified by each bacterial carrier (Kp (-); n = 8 patients, Kp (+); n = 37 patients, Pm (-); n = 37 patients, Pm (+); n = 8 patients, Eg (-); n = 11 patients, Eg (+); n = 34 patients). Data represent the mean ± SEM. The two-sided Mann-Whitney test was applied. ns: not significant.

Supplementary Figure 2

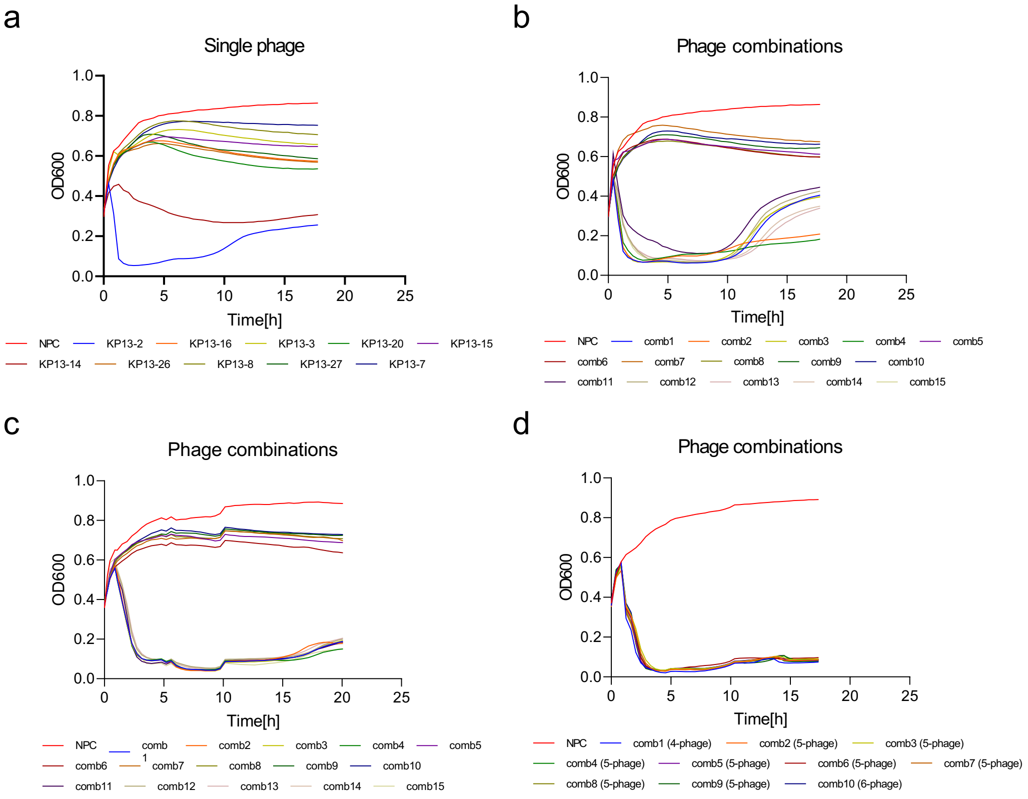

Supplementary Figure 2. Phage combinations against Kp derived from patients with PSC efficiently suppressed bacterial growth in vitro. a-d Testing the in vitro-growth suppression by single phages (**a**) and phage combinations (**b-d**) against Kp-P1. Bacterial growth was assessed by determining the OD_{600} of 1.2 over a 20 h period. The results obtained with the final optimized phage cocktail are shown in (**d**). Each plot shows the median of three biologically independent samples.

Supplementary Figure 3

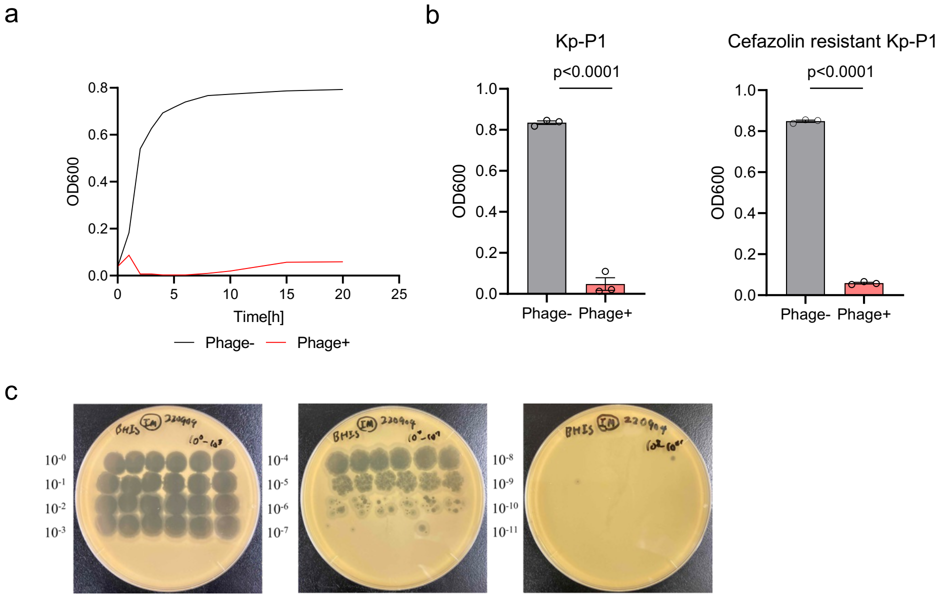

Supplementary Figure 3. Phage cocktail was effective in suppressing the growth of cefazolin resistant Kp-P1. a Testing the in vitro-growth inhibition by a finally optimized phage cocktail against cefazolin resistant Kp-P1. Bacterial growth was assessed by OD_{600} of 0.2 over a 20 h period. **b** In vitro growth suppression by the phages combination against Kp-P1 (n = 3 biologically independent samples per group; left) and cefazolin resistant Kp-P1(n = 3 biologically independent samples per group; right) at 20 h. **c** Representative photo of the plaque assay demonstrating the bacteriolysis by stepwise diluted concentrations of the selected phage cocktail. Data represent the mean ± SEM. The two-sided Student's t-test was applied.

Supplementary Figure 4

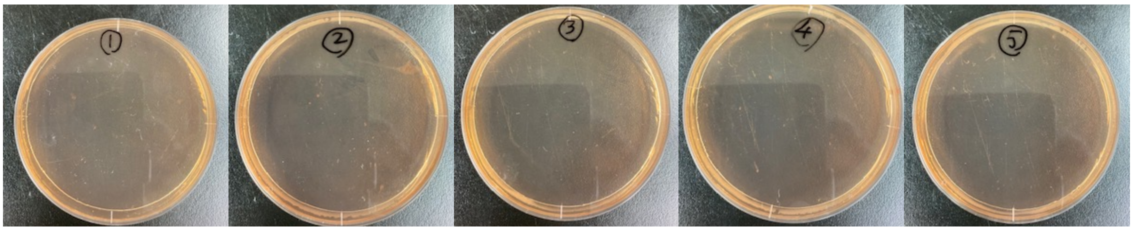

Supplementary Figure 4. Kp was not detected in the liver by culture. Livers of SPF mice colonized with Kp-P1 for 14 days pretreated with AMPC 7 days before inoculation with Kp-P1. One lobe of the liver was homogenized using BioMasherII (Nippi, Japan) with 500 µL of sterile PBS. 100µL of the sample was spotted on the deoxycholate-hydrogen sulfide-lactose agar (Nissui, Japan) and incubated at 37 °C for 24 hours (n = 5 mice).

Supplementary Figure 5

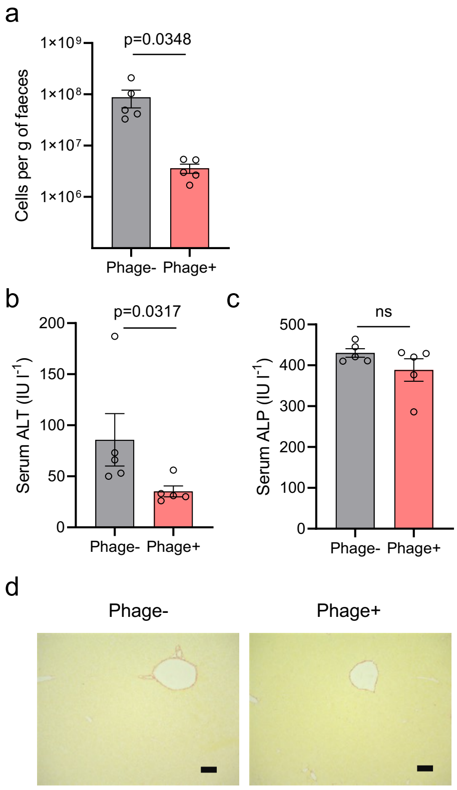

Supplementary Figure 5. Kp inoculation alone did not induce PSC-like hepatobiliary injuries in SPF mice. a Kp levels in faecal samples on the 28th day after colonization (n = 5 mice per group). **b, c** Serum ALT (**b**) and ALP levels (**c**) on the 14th day after colonization (n = 5 mice per group). **d** Representative photomicrographs of Sirius Red staining and of the liver sections of mice on the 14th day after colonization. Scale bars, 100μm. Data represent the mean ± SEM. The two-sided Student's t-test (for **a** and **c**) or the two-sided Mann–Whitney test (for **b**) was applied. ns : not significant.

Supplementary Figure 6

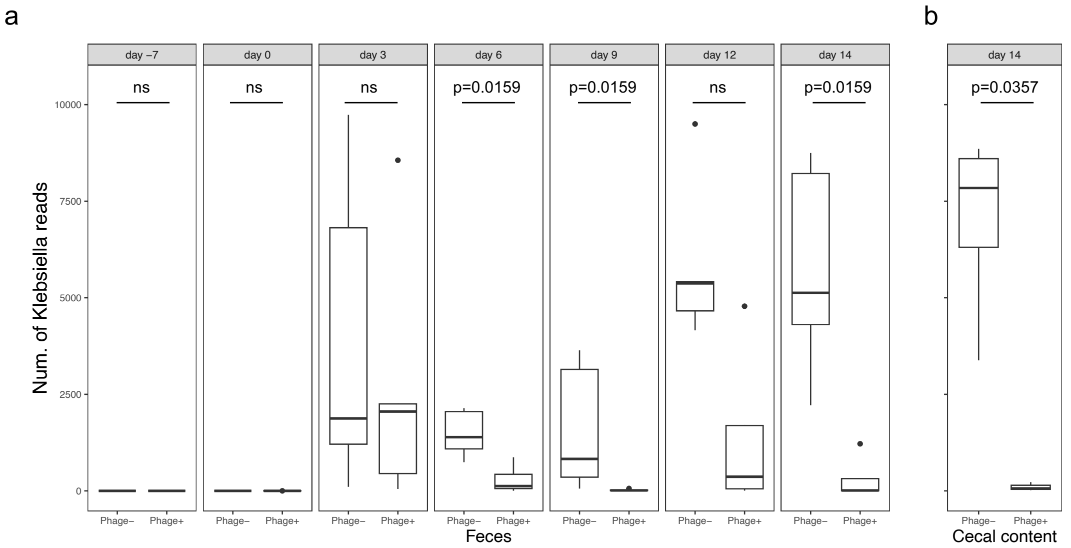

Supplementary Figure 6. The abundance of *Klebsiella* spp. a, b Boxplots showing the number of Klebsiella reads in faecal (**a**) and caecal (**b**) samples collected at the indicated time points. The numbers of biologically independent samples are as follows: for faecal samples, 3 phage- and 4 phage+ samples on day -7, 4 phage- and 4 phage+ samples on day 0, 5 phage- and 5 phage+ samples on days 3, 6, and 9, and 5 phage- and 4 phage+ samples on days 12 and 14. For caecal samples, there were 5 phage- and 3 phage+ samples on day 14. The two-sided Wilcoxon rank-sum exact test as applied. ns: not significant. Boxes extend from 25th to 75th percentiles. The lines within boxes are the median values. Whiskers represent the maximum and minimum values. Dots indicate the outliers.

Supplementary Figure 7

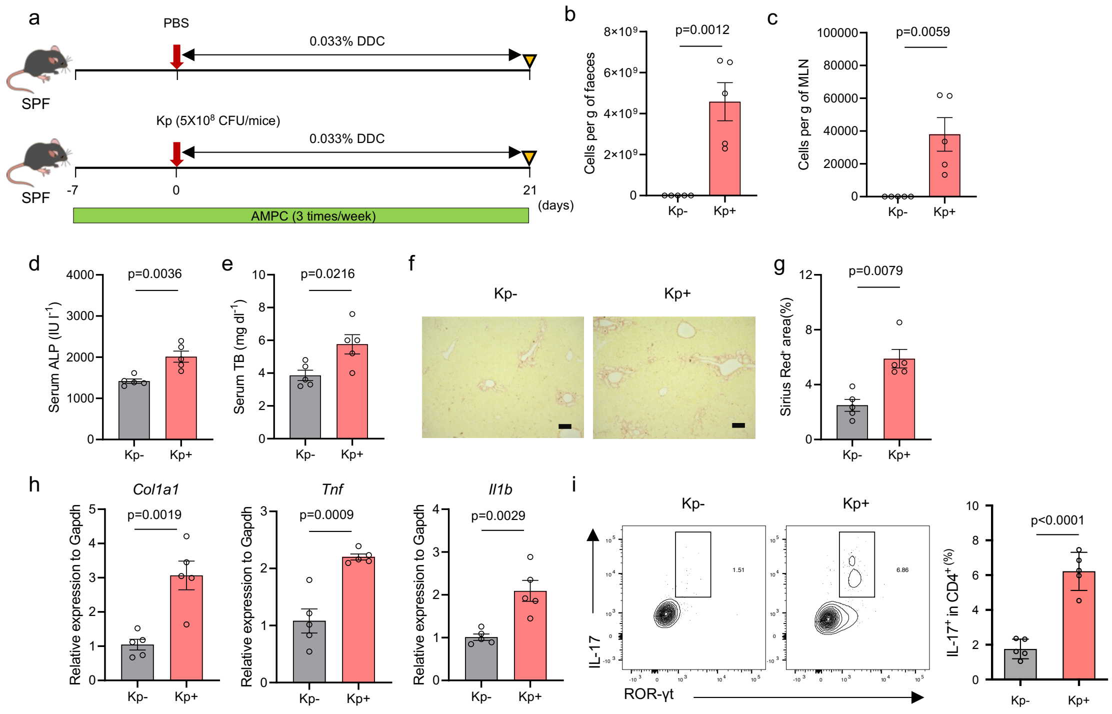

Supplementary Figure 7. Single Kp inoculation worsened DDC-induced hepatobiliary inflammation and fibrosis progression. a Study design: SPF mice were pretreated with AMPC 7 days before being inoculated with patient-derived Kp or PBS, followed by DDC feeding for three weeks (n = 5 mcie per group). **b, c** Amount of Kp in faecal samples (**b**) and in MLNs (**c**) assessed by qPCR. **d, e** Serum ALP levels (**d**) and TB levels (**e**) of mice. **f** Representative photomicrographs of Sirius Red staining and of the liver sections of mice. Scale bars, 100μm. **g** Quantitative Sirius Red-positive area of the liver sections of mice. **h** qRT-PCR analysis of *Col1a1, Tnf and Il1b* relative to *Gapdh* in the whole liver of mice. The numbers in each graph indicate the ratio compared to the vehicle group. **i** The representative FACS staining of intracellular IL-17 and ROR-γt in the liver (left) and the frequency of IL-17^{+} in CD4+ T cells (right). Data represent the mean ± SEM. The two-sided Student's t-test (for **b**, **c**, **d**, **e**, **h,** and **i**) or the two-sided Mann–Whitney test (for **g**) was applied. Data are representative of two independent experiments.

Supplementary Figure 8

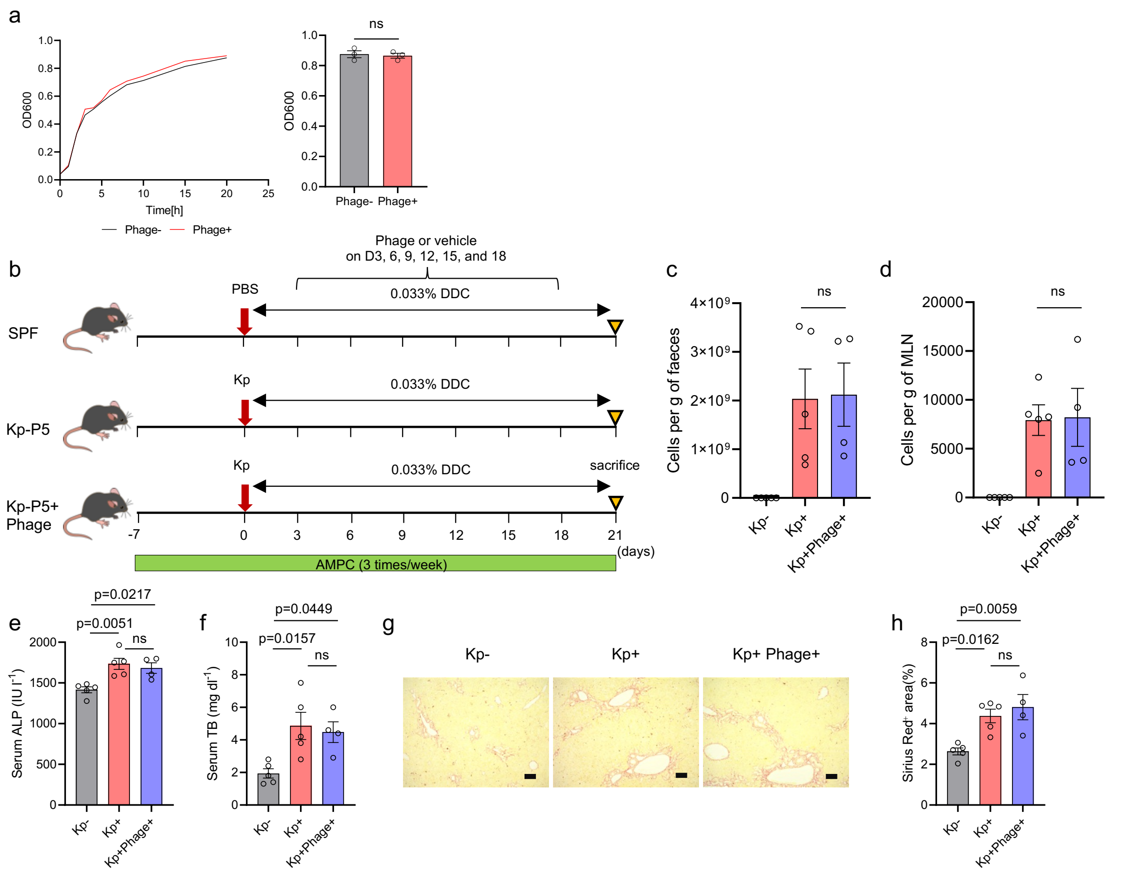

Supplementary Figure 8. Kp-P5 inoculation worsened DDC-induced hepatobiliary inflammation and fibrosis progression. a Testing the in vitro-growth inhibition by the finally optimized phage cocktail against Kp-P5. Bacterial growth was assessed by OD_{600} of 0.2 over a 20 h period (left). In vitro growth suppression by the phage cocktail against Kp-P5 (n = 3 biologically independent samples per group) at 20 h (right). **b** Study design: SPF mice were pretreated with AMPC 7 days before being inoculated with PBS, Kp-P5, or bacteriophage followed by DDC feeding for three weeks (Kp- : n = 5 mice, Kp+Phage- : n = 5 mice, Kp+Phage+ : n = 4 mice). **c**, **d** Amount of Kp in faecal samples (**c**) and in MLNs (**d**) assessed by qPCR. **e, f** Serum ALP levels (**e**) and TB levels (**f**) of mice. **g** Representative photomicrographs of Sirius Red staining and of the liver sections of mice. Scale bars, 100μm. **h** Quantitative Sirius Red-positive area of the liver sections of mice. Data represent the mean ± SEM. The two-sided Student's t-test (for **a**, **c**, and **d**) or one-way ANOVA with Tukey's multiple-comparison test (for **e**, **f**, and **h**) was applied. ns : not significant.

Supplementary Figure 9

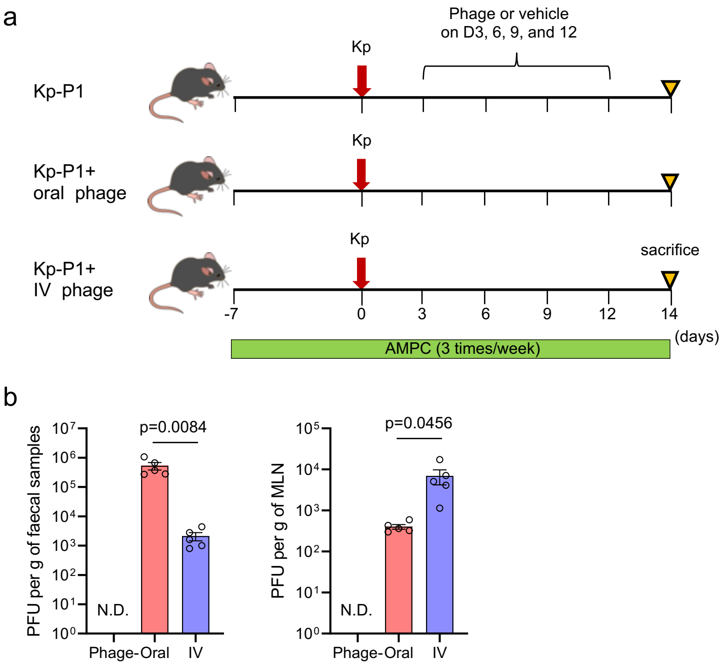

Supplementary Figure 9. Intravenous (IV) administration of phages achieved a superior effect on the delivery of phages to the MLN. a Study design: SPF mice were pretreated with AMPC 7 days before inoculation with patient-derived Kp and then administered either the vehicle or phage cocktail three times per week for 2 weeks (n = 5 mice per group). **b** Phage levels in faecal samples (left) and MLNs (right). N.D. : not detected. Data represent the mean ± SEM. The two-sided Student’s t-test was applied.

Supplementary Table 1. Characteristics of study population

	PSC(n=45)
Gender, male: female	28:17
Age, years<br>median (min-max)	36(13-67)
Observation period, years<br>median (min-max)	7.4(1.4-29.6)
ALT, IU/L<br>median (min-max)	50(9-190)
ALP, IU/L<br>median (min-max)	541(194-2525)
TB, mg/dL<br>median (min-max)	0.8(0.4-23.7)
PLT, 10^4/μL<br>median (min-max)	24.8(6.0-48.1)
CRP, mg/dL<br>median (min-max)	0.44(0.01-4.33)
Presence of intestinal lesion	34/45(76%)
CD/UC/IBD unclassified	1/16/17
Clinical outcome<br>(Alive/Dead/LTx)	37/6/2

LTx; Liver transplantation

Supplementary Table 2. Characteristics of study population

	none	Kp+Eg	p Value * # (none vs. Kp+Eg)
Gender, male: female	9:8	19:9	NS
Age, years median (min-max)	42	33	NS
Observation period, years median (min-max)	7.1	8.0	NS
ALT, IU/L median (min-max)	30	57.5	0.0246
ALP, IU/L median (min-max)	381	677	0.0075
TB, mg/dL median (min-max)	0.8	1.0	0.0245
PLT, 10^4/μL median (min-max)	24.8	24.9	NS
CRP, mg/dL median (min-max)	0.14	0.59	0.0238
Presence of intestinal lesion	12/17 (71%)	22/28 (76%)	NS
Clinical outcome (Alive/Dead or LTx)	17/0	20/8	0.0171

* Fisher's exact test was used to compare gender distribution, presence of intestinal lesion, and clinical outcome.

# The two-sided Mann–Whitney test was used to compare between PSC/UC and UC patients.

Supplementary Table 3. Information of bacteriophage

Phage	species	genus	family	genome size
KP13-2	novel	Webervirus	Drexlerviridae	49373
KP13-16	novel	Phapecoctavirus	Stephanstirmvirinae (subfamily)	137240
KP13MC5-1	novel	Slopekvirus	Straboviridae	174200
KP13MC5-2	novel	Teetrevirus	Autographiviridae	39447

Supplementary Table 4. Genetic characteristics of Kp strains used in this study

	number_genes	GC	genome_size	AMR_genes	virulence_genes#
mean*	5152.78	57.05	5579846.86	9.92	6.43
std*	432.75	0.54	488095.28	4.70	6.09
Kp-p1	5144	57.19	5556871	6	8
Kp-p1 z-score	-0.02	0.26	-0.05	-0.83	0.26
Kp-p5	5090	57.18	5470266	6	3
Kp-p5 z-score	-0.15	0.24	-0.22	-0.83	-0.56

*calculated from 100 Kp strains isolated from PSC patients.

downloaded from uniport all proteins with (origin=kp) + (keyword=virulence):
https://www.uniprot.org/uniprotkb?query=(organism_id:573)%20AND%20(keyword:KW-0843)

Supplementary Table 5. Pre-designed primers used for quantitative real-time polymerase chain reaction

Primer	Nucleotide Sequence (forward)	Nucleotide Sequence (reverse)	Catalog number
Col1a1	5'-GACATGTTCAGCTTTGTGGA-3'	5'-GGGACCCTTAGGCCATTGTGTA-3'	MA1107374
Tnf	5'-AAGCCTGTAGCCCACGTCGTA-3'	5'-GGCACCACTAGTTGGTTGTCTTTG-3'	MA031450
Il1b	5'-TCCAGGATGAGGACATGAGCAC-3'	5'-GAACGTCACACACCAGCAGGTTA-3'	MA025939
Gapdh	5'-TGCACCACCAACTGCT-3'	5'-GGATGCAGGGATGATG-3'	1622366
