# Supplementary material for: Bacteriophage therapy against pathological Klebsiella pneumoniae ameliorates the course of primary sclerosing cholangitis
Source: Nat Commun. 2023 Jun 5;14:3261. doi: 10.1038/s41467-023-39029-9 (PMC10241881; doi:10.1038/s41467-023-39029-9)
Supplement: Supplementary file 2 — Description of Additional Supplementary Files [file 41467_2023_39029_MOESM2_ESM.docx]

**Description of Additional Supplementary Files**

File Name: Supplementary Data 1

Description: List of bacteriophages combination

File Name: Supplementary Data 2

Description: Taxonomic assignment for ASVs

File Name: Supplementary Data 3

Description: ASV counts and statistical results

File Name: Supplementary Data 4

Description: Read counts of genera with significant difference in the abundance between phage -(neg)/ +(pos) groups at Day14
