# Supplementary material for: Bacteriophage therapy against pathological Klebsiella pneumoniae ameliorates the course of primary sclerosing cholangitis
Source: Nat Commun. 2023 Jun 5;14:3261. doi: 10.1038/s41467-023-39029-9 (PMC10241881; doi:10.1038/s41467-023-39029-9)
Supplement: Supplementary file 7 — Reporting summary [file 41467_2023_39029_MOESM7_ESM.pdf]

nature portfolio

Corresponding author(s): Nobuhiro Nakamoto, Takanori Kanai

Last updated by author(s): May 15, 2023

Reporting Summary

Nature Portfolio wishes to improve the reproducibility of the work that we publish. This form provides structure for consistency and transparency in reporting. For further information on Nature Portfolio policies, see our Editorial Policies and the Editorial Policy Checklist.

Statistics

For all statistical analyses, confirm that the following items are present in the figure legend, table legend, main text, or Methods section.

n/a	Confirmed	
☐	☒	The exact sample size (*n*) for each experimental group/condition, given as a discrete number and unit of measurement
☐	☒	A statement on whether measurements were taken from distinct samples or whether the same sample was measured repeatedly
☐	☒	The statistical test(s) used AND whether they are one- or two-sided<br>*Only common tests should be described solely by name; describe more complex techniques in the Methods section.*
☐	☒	A description of all covariates tested
☐	☒	A description of any assumptions or corrections, such as tests of normality and adjustment for multiple comparisons
☐	☒	A full description of the statistical parameters including central tendency (e.g. means) or other basic estimates (e.g. regression coefficient) AND variation (e.g. standard deviation) or associated estimates of uncertainty (e.g. confidence intervals)
☐	☒	For null hypothesis testing, the test statistic (e.g. *F*, *t*, *r*) with confidence intervals, effect sizes, degrees of freedom and *P* value noted<br>*Give P values as exact values whenever suitable.*
☒	☐	For Bayesian analysis, information on the choice of priors and Markov chain Monte Carlo settings
☒	☐	For hierarchical and complex designs, identification of the appropriate level for tests and full reporting of outcomes
☒	☐	Estimates of effect sizes (e.g. Cohen's *d*, Pearson's *r*), indicating how they were calculated

Our web collection on statistics for biologists contains articles on many of the points above.

Software and code

Policy information about availability of computer code

Data collection	BD FACSDiva version 8.0.1 for flowcytometry.
Data analysis	FlowJo v10 (Flow Jo LLC) for flowcytometry, Prism v8(Graphpad) for the statistics, ImageJ 1.5.2a for quantitative analysis of the fibrotic area, DADA2 pipeline (v1.18.0) and GLSEARCH program for 16S rRNA analysis, and SPAdes 3.10.1, PhageTerm 1.0.12, PATRIC, BLAST, the NCBI AMRfinder tool, PFAM version 33.1, HMMER version 3.3, PHACTS v0.3, and BACPHLIP v0.9.3 for phage bioinformatic analyses.

For manuscripts utilizing custom algorithms or software that are central to the research but not yet described in published literature, software must be made available to editors and reviewers. We strongly encourage code deposition in a community repository (e.g. GitHub). See the Nature Portfolio guidelines for submitting code & software for further information.

Data

Policy information about availability of data

All manuscripts must include a data availability statement. This statement should provide the following information, where applicable:

- Accession codes, unique identifiers, or web links for publicly available datasets
- A description of any restrictions on data availability
- For clinical datasets or third party data, please ensure that the statement adheres to our policy

16S rRNA sequencing and whole-genome sequencing data have been deposited in the DDBJ database with accession numbers DRA015605 (https://ddbj.nig.ac.jp/resource/sra-submission/DRA015605) for 16S rRNA sequencing, PRJNA887913 (https://www.ncbi.nlm.nih.gov/bioproject/PRJNA887913) for the whole-genome sequencing of Klebsiella phages, and PRJDB7545 (https://www.ncbi.nlm.nih.gov/search/all/?term=PRJDB7545) for the whole-genome sequencing of Klebsiella

pneumoniae strains used in this study. Source data are provided with this paper.

Human research participants

Policy information about studies involving human research participants and Sex and Gender in Research.

Reporting on sex and gender	Sex and gender determined based on self-reporting were not considered in the study design. Gender-based analyses were not performed because gender differences are not expected to have a significant impact on the results of this study.
Population characteristics	45 patients with PSC complicated with IBD (n = 34) or without IBD (n = 11) were included in this study. PSC was diagnosed according to clinical guidelines and typical cholangiography findings (endoscopic retrograde cholangiography and/or magnetic resonance cholangiopancreatography) or liver biopsy. All patients underwent at least one colonoscopy to exclude the presence of IBD. The IBD subtype was classified according to standard disease descriptions based on a combination of endoscopic, histopathological, radiological, and serological investigations<br>Clinical characteristics are presented in Supplementary Table 1.
Recruitment	Human research participants were recruited at Keio University Hospital. Written informed consent was obtained from all subjects. In the case of minors, written informed consent was obtained from their parents. There is no indication that self selection bias affected any of the results.
Ethics oversight	The ethics committee at Keio University School of Medicine approved the study protocol (approval #20140211) in accordance with the principles of Helsinki Declaration II.

Note that full information on the approval of the study protocol must also be provided in the manuscript.

Field-specific reporting

Please select the one below that is the best fit for your research. If you are not sure, read the appropriate sections before making your selection.

☒ Life sciences ☐ Behavioural & social sciences ☐ Ecological, evolutionary & environmental sciences

For a reference copy of the document with all sections, see nature.com/documents/nr-reporting-summary-flat.pdf

Life sciences study design

All studies must disclose on these points even when the disclosure is negative.

Sample size	No statistical methods were used to predetermine sample size. Sample sizes were determined by allowable error size, accuracy, resources, and need for statistical analysis based on previous studies from our group (J Clin Invest. 2018 Apr 2;128(4):1581-1596, Nat microbiol. 2019 4 (3): 492-503, J Hepatol. 2021 Mar;74(3):511-521.) and publications in the field.
Data exclusions	No data were excluded from the analysis.
Replication	Experiments were replicated several times with reproducible results, as indicated in each figure legend.
Randomization	All animals used were age, gender and vendor matched. Animals were randomly allocated to each group.
Blinding	The data collection was not blinded. Blinding was not possible as the investigators were also conducting the experiments and had to be aware of controls and treated groups.

Reporting for specific materials, systems and methods

We require information from authors about some types of materials, experimental systems and methods used in many studies. Here, indicate whether each material, system or method listed is relevant to your study. If you are not sure if a list item applies to your research, read the appropriate section before selecting a response.

Materials & experimental systems

n/a	Involved in the study
	☒ Antibodies
☒	Eukaryotic cell lines
☒	Palaeontology and archaeology
	☒ Animals and other organisms
☒	Clinical data
☒	Dual use research of concern

Methods

n/a	Involved in the study
☒	ChIP-seq
	☒ Flow cytometry
☒	MRI-based neuroimaging

Antibodies

Antibodies used

For flow cytometry analysis, the following antibodies were used in this study. Anti-FcR (CD16/32, BD Pharmingen), anti-mouse anti-TCRb (no.109228, Biolegend, PerCP/Cy5.5 conjugate, clone H57-597), anti-CD3e (no.552774, BD bioscience, PE-cy7 conjugate, clone 145-2C11), anti-CD4 (no.563106, BD bioscience, BV510 conjugate, clone RM4-5), Fixable Viability Dye eFluor780 (no.65-0864-14, eBioscience), anti-IFN-g (no.554412, BD bioscience, PE conjugate, clone XMG1.2), anti-IL-17A (no.560221, BD Bioscience, Alexa Flour488 conjugate, clone TC11-18H10), anti-IL-22 (no.17-7222-82, eBioscience, APC conjugate, clone IL22JOP), and anti-RORgt (no.562894, BD bioscience, BV421 conjugate, clone Q31-378).

Validation

All the antibodies used in this study were commercial antibodies and were only used for applications, with validation procedures described on the sites of the manufacturers.
https://www.thermofisher.com; https://www.biolegend.com; https://www.bdbiosciences.com.

Anti-FcR (CD16/32, BD Pharmingen) https://www.bdbiosciences.com/en-us/products/reagents/flow-cytometry-reagents/research-reagents/single-color-antibodies-ruo/purified-rat-anti-mouse-cd16-cd32.567021
Anti-mouse anti-TCRb (no.109228, Biolegend, PerCP/Cy5.5 conjugate, clone H57-597) https://www.biolegend.com/ja-jp/products/percp-cyanine5-5-anti-mouse-tcr-beta-chain-antibody-5603
Anti-CD3e (no.552774, BD bioscience, PE-cy7 conjugate, clone 145-2C11) https://www.bdbiosciences.com/en-us/products/reagents/flow-cytometry-reagents/research-reagents/single-color-antibodies-ruo/pe-cy-7-hamster-anti-mouse-cd3e.552774
Anti-CD4 (no.563106, BD bioscience, BV510 conjugate, clone RM4-5) https://www.bdbiosciences.com/ja-jp/products/reagents/flow-cytometry-reagents/research-reagents/single-color-antibodies-ruo/bv510-rat-anti-mouse-cd4.563106
Fixable Viability Dye eFluor780 (no.65-0864-14, eBioscience) https://www.thermofisher.com/order/catalog/product/jp/ja/65-0865-14
Anti-IFN-g (no.554412, BD bioscience, PE conjugate, clone XMG1.2) https://www.bdbiosciences.com/ja-jp/products/reagents/flow-cytometry-reagents/research-reagents/single-color-antibodies-ruo/pe-rat-anti-mouse-ifn.554412
Anti-IL-17A (no.560221, eBioscience, Alexa Flour488 conjugate, clone TC11-18H10) https://www.bdbiosciences.com/en-au/products/reagents/flow-cytometry-reagents/research-reagents/single-color-antibodies-ruo/alexa-fluor-488-rat-anti-mouse-il-17a.560221
Anti-IL-22 (no.17-7222-82, eBioscience, APC conjugate, clone IL22JOP) https://www.thermofisher.com/antibody/product/IL-22-Antibody-clone-IL22JOP-Monoclonal/17-7222-82
Anti-RORgt (no.562894, BD bioscience, BV421 conjugate, clone Q31-378) https://www.bdbiosciences.com/ja-jp/products/reagents/flow-cytometry-reagents/research-reagents/single-color-antibodies-ruo/bv421-mouse-anti-mouse-ror-t.562894

Animals and other research organisms

Policy information about studies involving animals; ARRIVE guidelines recommended for reporting animal research, and Sex and Gender in Research

Laboratory animals

Male GF mice (C57BL/6 background strain, 6-8-weeks old) were purchased from Sankyo Lab Service Corporation (Tokyo, Japan) and kept in the GF Facility of Keio University School of Medicine. Male C57BL/6 mice (6-8-weeks old) were purchased from Japan CLEA (Tokyo, Japan) and maintained under SPF conditions. The mice were kept in the Central Laboratories for Experimental Animals (Kawasaki, Japan) and the Animal Care Facility of Keio University School of Medicine, with a 12-hour light/dark cycle, a temperature range of 22-25 °C, and a relative humidity of 45-55%.

Wild animals

This study did not involve wild animals.

Reporting on sex

Male mice were used for animal studies, since female hormones can affect the fibrosis progression resulting in variations.

Field-collected samples

This study did not involve samples collected from the field.

Ethics oversight

Animal Ethics Committee of Keio University approved all animal studies.

Note that full information on the approval of the study protocol must also be provided in the manuscript.

Flow Cytometry

Plots

Confirm that:

☒ The axis labels state the marker and fluorochrome used (e.g. CD4-FITC).

☒ The axis scales are clearly visible. Include numbers along axes only for bottom left plot of group (a 'group' is an analysis of identical markers).

☒ All plots are contour plots with outliers or pseudocolor plots.

☒ A numerical value for number of cells or percentage (with statistics) is provided.

Methodology

Sample preparation	Sample preparation is described in Methods section. Briefly, livers were perfused through the portal vein with phosphate-buffered saline, then minced and passed through 100 μm nylon mesh. The filtrate was centrifuged at 50g for 1 min and supernatant was washed once. Cells were suspended in a Histopaque solution (Sigma–Aldrich) and overlaid on a HBSS solution. After centrifugation at 2500 rpm for 20 min, the cells were collected from the upper face of the Histopaque. The numbers of live cells were determined by Countess II (Thermo Fisher Scientific).
Instrument	Isolated cells were stained with fluorochrome-conjugated antibody.<br>For analysis: BD FACSCanto™ II
Software	FACSDiva for collection and FlowJo (v10) for analysis
Cell population abundance	Populations were validated for purity by a post-sort analysis by FACS.
Gating strategy	Lymphocytes were determined by FSC-A/SSC-A, doublets were excluded by FSC-W/FSC-H, viable cells were gated by Fixble Viability Dye eFluor 780 nagative cells, CD3+ CD4+ events were gated, and each population was analyzed as described in the figures. Further gating strategies will be provided upon request.

☐ Tick this box to confirm that a figure exemplifying the gating strategy is provided in the Supplementary Information.
